# Endangered Père David’s deer genome provides insights into population recovering

**DOI:** 10.1111/eva.12705

**Published:** 2018-10-09

**Authors:** Lifeng Zhu, Cao Deng, Xiang Zhao, Jingjing Ding, Huasheng Huang, Shilin Zhu, Zhiwen Wang, Shishang Qin, Yuhua Ding, Guoqing Lu, Zhisong Yang

**Affiliations:** ^1^ College of life Sciences Nanjing Normal University Nanjing China; ^2^ University of Nebraska at Omaha Omaha; ^3^ DNA Stories Bioinformatics Center Chengdu China; ^4^ PubBio‐Tech Services Corporation Wuhan China; ^5^ Jiangsu Academy of Forestry Nanjing China; ^6^ Shanghai Majorbio Bio‐pharm Biotechnology Co. Ltd. Shanghai China; ^7^ Jiangsu Dafeng Milu National Nature Reserve Dafeng China; ^8^ Key Laboratory of Southwest China Wildlife Resources Conservation (ministry of education) China West Normal University Nanchong China

**Keywords:** breeding success, Père David's deer, population recovering, selective pressure, the high‐salt diet

## Abstract

The Milu (Père David's deer, *Elaphurus davidianus*) were once widely distributed in the swamps (coastal areas to inland areas) of East Asia. The dramatic recovery of the Milu population is now deemed a classic example of how highly endangered animal species can be rescued. However, the molecular mechanisms that underpinned this population recovery remain largely unknown. Here, different approaches (genome sequencing, resequencing, and salinity analysis) were utilized to elucidate the aforementioned molecular mechanisms. The comparative genomic analyses revealed that the largest recovered Milu population carries extensive genetic diversity despite an extreme population bottleneck. And the protracted inbreeding history might have facilitated the purging of deleterious recessive alleles. Seventeen genes that are putatively related to reproduction, embryonic (fatal) development, and immune response were under high selective pressure. Besides, *SCNN1A,* a gene involved in controlling reabsorption of sodium in the body, was positively selected. An additional 29 genes were also observed to be positively selected, which are involved in blood pressure regulation, cardiovascular development, cholesterol regulation, glycemic control, and thyroid hormone synthesis. It is possible that these genetic adaptations were required to buffer the negative effects commonly associated with a high‐salt diet. The associated genetic adaptions are likely to have enabled increased breeding success and fetal survival. The future success of Milu population management might depend on the successful reintroduction of the animal to historically important distribution regions.

## 1 INTRODUCTION

**Figure 1:**
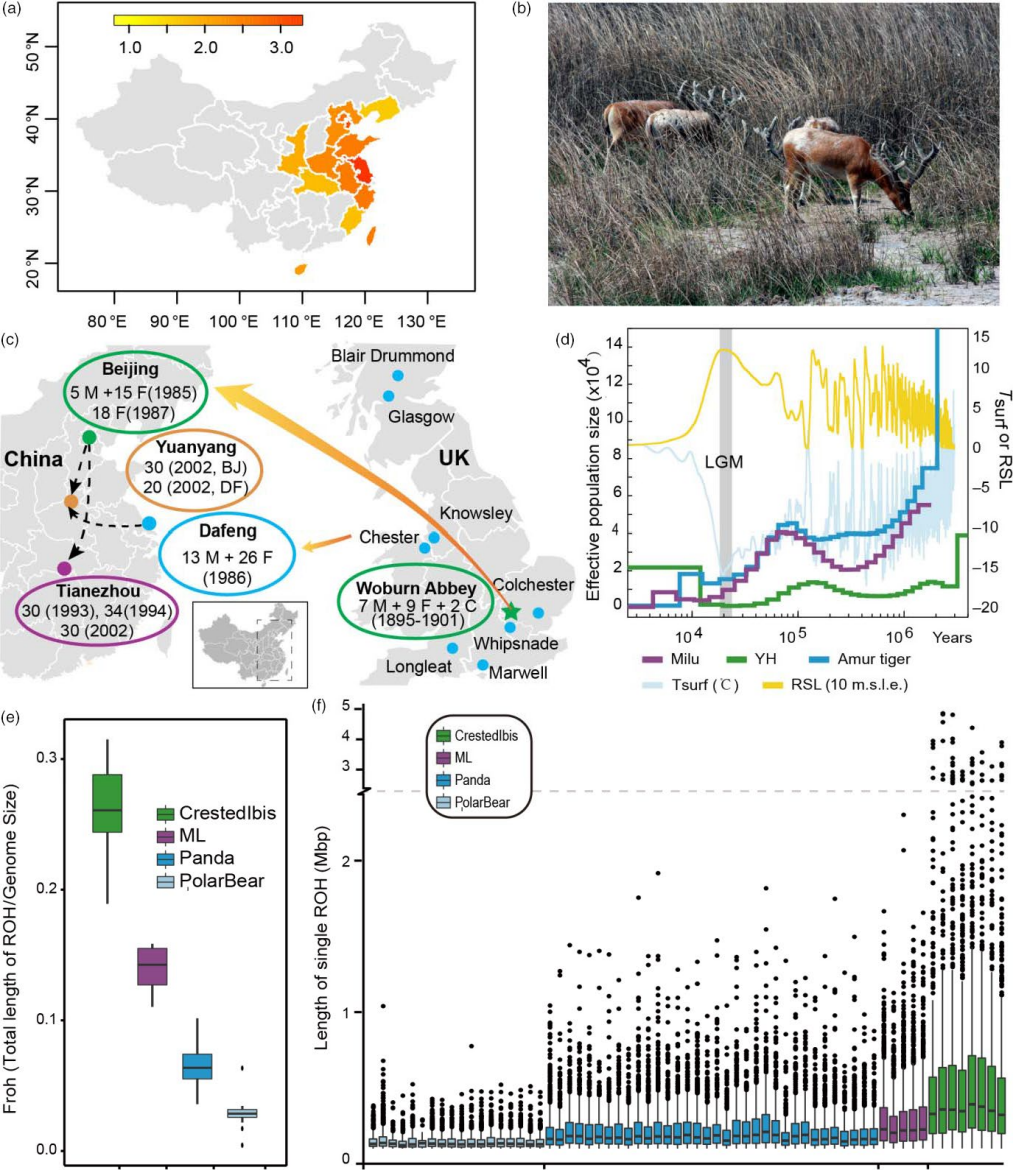
The history of the Milu. (a) Paleogeographic distribution history of wild Milu in China. The data for Milu fossils were adopted from Cao, 2005. The color relates to the density of the fossils in specific provinces, and the density was calculated as the number of fossils per million square kilometers. (b) Milu foraging in the coastal shoal habitat of Dafeng Milu Natural Reserve, Jiangsu, China. (c) Large‐scale reintroduction programs since 1985. (c) fawn; F, females; M, males. (d) Demographic history of the Milu. The history of the Milu population and climate change spans from 3 KYA to 4 MYA. We used the default mutation rate of 1.5 × 10^–8^ for baiji (μ) and an estimation of 6 years per generation (g). The last glacial maximum (LGM) is highlighted in gray. Tsurf, atmospheric surface air temperature; RSL, relative sea level; 10 m.s.l.e., 10 m sea level equivalent. (e) Box plot of Froh for Milu, crested ibis, panda, and polar bear populations. Froh denotes the proportion of total ROH length. (f) Box plot of length of ROH in each individual from Milu, crested ibis, panda, and polar bear

Milu was once widely distributed in the swamps of East Asia where they were predominantly found in China (Figure 1a, b and Supporting information Figure S1). This species was first introduced to the west in 1866 by Armand David (Père David) and subsequently became extinct in its native China in the early 20th century (Cao, 2005). Fortunately, between 1894 and 1901, Herbrand Arthur Russell (the 11th Duke of Bedford) acquired the few remaining deer (18 individuals) from European zoos. These individuals were nurtured at Woburn Abbey in England (Figure 1c), and the current world population was derived from this herd (Cao, 2005). In the mid‐1980 s, 77 individuals were reintroduced to captive facilities in China (Cao, 2005; Jiang & Harris, 2014), and populations were established in Beijing, Dafeng, Tianezhou, and Yuanyang (Figure 1c). The largest recovered Milu population in the world lives in the Dafeng coastal region. However, high‐salt diet‐related diseases have not been observed over the last several decades in this high‐salt area. Since then, the populations have rapidly expanded. The repopulation of Milu has now deemed a classic example of how a highly endangered species can be rescued. However, the molecular mechanisms that underpinned this population recovery remain largely unknown.

Survival and reproduction are important properties of all living organisms, and Milu is no exception in this regard. Currently, the largest recovered Milu population in the world lives in the Dafeng coastal district (this population increased from 39 to over 3,000 individuals between the 1980s and 2017). This region is historically synonymous with the Milu (Cao, 2005). Although the foundation population size was initially very small, the recovering population does not exhibit any adverse effects (such as high juvenile mortality) usually associated with potential inbreeding depression. From 1986 to 2002, the mean birth rate was approximately 25% (Ding, 2017); this rate approximates to that of the wild white‐tailed deer (*Odocoileus virginia*) (21%–22%) (Stoll Jr & Parker, 1986). The juvenile mortality rate in the Dafeng population was approximately 5% between 1986 and 2002 (Ding, 2017), which is far lower than that exhibited by other captive mammalian populations (both non‐inbred and inbred populations) (O'Brien et al., 1985; Zeng, Jiang, & Li, 2007). For example, the juvenile mortality rate of an inbred cheetah population was reported to be 29.1% (O'Brien et al., 1985). Previous research also revealed that juvenile mortality was higher in inbred populations than in non‐inbred populations in 15 out of 16 captive ungulate species. This characteristic of inbred populations is most likely caused by the deleterious effects associated with inbreeding (Zeng et al., 2007). In this study, we put forward two hypotheses as to how Milu survived potential inbreeding depression characteristics. Hypothesis I contests that the founding population of resettled Milu did not contain deleterious recessive alleles that cause increased mortality often associated with inbreeding depression. Furthermore, the Milu mating system is polygynous (Chunwang, Zhigang, Yan, & Caie, 2004), which means that a single male breed with many females. Similar to the situation in gorillas, this characteristic means that spermatogenesis or sperm fertility is vitally important (Dixson, 1998). This situation has resulted in a relatively high birthrate and a very low juvenile mortality rate in the Milu population. Hypothesis II is that the Milu has experienced putative selective pressures on genes associated with reproduction, embryonic development, and immune response Milu.

Previous genetic studies using mitochondrial and microsatellite loci have helped to investigate the population genetics of the Milu. These analyses have revealed that the Milu exhibit extremely low genetic diversity caused by reduced foundation population sizes (Zeng et al., 2007). The challenge of studying the genetic characteristics of Milu populations has been aided by the introduction of next‐generation sequencing technologies. The relatively inexpensive generation of large volumes of sequence data is a big advantage in comparison with conventional methods. It is hoped that the generation of these data will help to address many biological questions relating to the Milu population recovery (Davey et al., 2011; DePristo et al., 2011; Metzker, 2010). In this study, different approaches (de novo genome sequencing and resequencing, and dietary analysis) were utilized to test the aforementioned hypotheses.

## 2 MATERIALS AND METHODS

### 2.1 Subjects

Twenty millitre blood sample was collected from one adult female Milu individuals in Dafeng Milu Natural Reserve in 2012, and 5 ml blood per sample was collected randomly from 5 adult Milu (3 females and two males) in Dafeng Milu Natural Reserve in 2013. The blood samples were deposited in vacuum blood collection tube (Heparin anticoagulation) and shipped to the laboratory by dry ices.

### 2.2 Genome sequencing and assembly

An adult female Milu in Dafeng Milu Natural Reserve was used for de novo sequencing. Samples from an additional five animals were utilized for resequencing. Genomic DNA was extracted using Puregene Tissue Core Kit A (Qiagen). Libraries with different insert sizes were constructed at Majorbio (Shanghai), and the insert sizes of the libraries were 180, 500, 800 bp, 3, 5, 8, and 10 kb. For short‐insert libraries (180–800 bp), 6 μg of DNA was fragmented to the desired insert size, end‐repaired, and ligated to Illumina paired‐end adaptors. Ligated fragments were size selected at 180, 500, and 800 bp on agarose gels and were purified by PCR amplification to yield the corresponding libraries. For long insert sizes (3, 5, 8, and 10 kb) mate‐pair library construction, 60 μg of genomic DNA was used; we circularized DNA, digested linear DNA, fragmented circularized DNA and purified biotinylated DNA, and then performed adaptor ligation. The libraries were sequenced using a HiSeq2000 instrument. The other five resequencing samples were sequenced with read and insert lengths of 101 and 500 bp, respectively.

Whole‐genome shotgun assembly of the Milu was performed using the short oligonucleotide analysis package, SOAP*denovo* (Li et al., 2010) with main parameters “*‐K 63 ‐d 1 ‐D 1 ‐R ‐F*.” After filtering the reads, short‐insert size library data were used to construct a *de Bruijn* graph without paired‐end information. Contigs were constructed by merging the bubbles and resolving the small repeats. All qualified reads were realigned to contig sequences and paired‐end relationships between the reads of allowed linkages between the contigs. We subsequently used the relationships, step‐by‐step, from the short‐insert size paired‐ends and the long‐distance paired‐ends to construct scaffolds. Gaps were then closed using the paired‐end information to retrieve read pairs in which one end mapped to a unique contig and the other was located in the gap region. Assembly quality was assessed by aligning the assembled WTD (Malenfant, Davis, Moore, & Coltman, 2014) and CSD (Yao, Zhao, Wang, et al., 2012; Yao, Zhao, Zhang, et al., 2012) transcripts with the Milu scaffolds and by BUSCO v3 (Simão, Waterhouse, Ioannidis, Kriventseva, & Zdobnov, 2015) (Supporting information Table S19).

### 2.3 Genome annotation

Transposable elements in the Milu genome were identified by a combination of homology‐based and de novo approaches**.** Tandem repeats were identified using Tandem Repeat Finder (Benson, 1999). Interspersed repeats were characterized by homolog‐based identification using RepeatMasker open‐4.0.3 (Smit, Hubley, & Green, 1996) and the repeat database, Repbase2. Repeated proteins were identified using RepeatProteinMask and the transposable elements protein database. De novo identified interspersed repeats were annotated using RepeatModeler (Price, Jones, & Pevzner, 2005), and LTR_FINDER (Xu & Wang, 2007) was used to identify the LTRs; these results were used to generate the de novo repeat libraries, and then RepeatMasker was run once more against the de novo libraries. All repeats identified in this manner were included in the total count of interspersed repeats.

The Milu protein‐coding genes were annotated following the use of a combination of homolog gene prediction and de novo gene prediction tools. For homolog gene prediction, the protein sequences from cow, yak, goat, TA, and human were mapped to the genome using tBLASTn (Altschul, Gish, Miller, Myers, & Lipman, 1990). GeneWise (Birney, Clamp, & Durbin, 2004) was used to predict the gene model based on the alignment results. De novo gene prediction was performed using GENSCAN (Burge & Karlin, 1997), AUGUSTUS (Stanke et al., 2006), and GLIMMERHMM (Majoros, Pertea, & Salzberg, 2004) based on the repeat‐masked genome. Then, EVM (Haas et al., 2008) and MAKER (Cantarel et al., 2008) were applied to integrate the predicted genes. Finally, manual integration was performed to construct the final gene set. We searched the final gene set against the KEGG (Kanehisa & Goto, 2000), SwissProt (Bairoch & Apweiler, 2000), and TrEMBL (Bairoch & Apweiler, 2000) protein databases to identify gene functions. The gene motifs and domains were determined using InterProScan (Zdobnov & Apweiler, 2001) following analysis of public protein databases, including ProDom, PRINTS, PFAM, SMART, PANTHER, and PROSITE. All genes were aligned against the KEGG pathway database (Kanehisa & Goto, 2000), and the best match for each gene was identified. The GO IDs for each gene were obtained from the corresponding InterPro entries. We also mapped Milu proteins to the NCBI nr database and retrieved GO IDs using BLAST2GO (Conesa et al., 2005).

### 2.4 Detection of variants

For the individual that was used for de novo sequencing, we used the BWA (Li & Durbin, 2009) program to remap the paired‐end (180, 500, and 800 bp) clean reads to the assembled scaffolds. After merging the BWA results, sorting alignments (using the leftmost coordinates), and removing potential PCR duplicates, we used SAMtools (Li et al., 2009) mpileup to call SNPs and short InDels. We applied vcfutils.pl varFilter (in SAMtools) as the filtering tool with parameters “*‐Q 20 ‐d 6 ‐D 86*.” Then, homologous SNP positions were extracted and further filtered, to disqualify SNPs that may have resulted from errors due to assembly and/or mapping. The heterozygosity rate was estimated as the density of heterozygous SNPs for the whole genome, gene intervals, introns, and exons, respectively. For the five resequencing Milu individuals (coverage: 11–14, Supporting information Table S1), variants were identified using similar methods, except that the filtering parameter used by vcfutils.pl varFilter was “*‐Q 20 ‐d 6 ‐D 75*” considering their low coverages.

Whole‐genome resequencing data from 34 giant panda genomes (Zhao et al., 2013) and eight crested ibis (Li et al., 2014) genomes were downloaded from the NCBI SRA database, and BAM files were generated using identical methods to those used for Milu individuals. Next, the bam files for each species were processed using the mpileup module in samtools and the following parameters; “‐q 1 ‐C 50 ‐g ‐t DP, SP, DP4 ‐I ‐d 250 ‐L 250 ‐m 2 ‐p.” The associated variants were called and filtered using the varFilter module of vcfutils.pl (parameters “*‐Q 20 ‐d 10 ‐D 50,000 –w 5 ‐W 10*” for panda, and “‐Q 20 ‐d 5 ‐D 4,000 ‐w 5 ‐W 10” for crested ibis). Finally, variants from each individual were generated by filtering positions with low depth (“<3” for panda, and “<5” for crested ibis). The SNP positions in 18 polar bear genomes (Liu et al., 2014) were extracted from variant files downloaded from GigaDB (Sneddon, Li, & Edmunds, 2012). SNPs were annotated using snpEff (Cingolani et al., 2012). To estimate how the functional changes for proteins in Milu/panda/polar bear/crested ibis differed from those in humans, we evaluated the likely effect of a mutation in humans relative to the Milu/panda/polar bear/crested ibis alleles as either neutral or deleterious using SIFT(Ng & Henikoff, 2003).

### 2.5 Demographic history reconstruction and ROH identification

Demographic histories of the Milu were reconstructed using the Pairwise Sequentially Markovian Coalescent (PSMC) model (Li & Durbin, 2011) with the BAM files from the individual that was used for de novo sequencing.The mutation rate (μ) was set to 1.5 × 10^−8^ and the generation time (g) was set to 6 years. Before ROH analysis, we used VCFtools (v0.1.12b) (Danecek et al., 2011) to evaluate the relatedness of these five resequencing individuals and found most of them were unrelated (Table 1). And then, we can identify the ROH for each individual using the runs of homozygosity tool in PLINK (v.1.07) (Purcell et al., 2007) with adjusted parameters (‐‐homozyg‐window‐kb 0 ‐‐homozyg‐window‐snp 65 ‐‐homozyg‐window‐het 1 ‐‐homozyg‐window‐missing 3 ‐‐homozyg‐window‐threshold 0.05 ‐‐homozyg‐snp 65 ‐‐homozyg‐kb 100 ‐‐homozyg‐density 5,000 ‐‐homozyg‐gap 5,000). The individual genome‐based inbreeding coefficient, denoted as *Froh*, is defined as the fraction of total ROH length to genome effective length (Gazal et al., 2014).

**Table 1:** Milu whole‐genome assembly statistics

	Scaffold		Contig	
	Length (bp)	Number	Length (bp)	Number
Max length	19,425,686	–	319,828	–
N10	7,474,043	27	94,849	2,057
N20	5,942,968	66	69,060	5,209
N30	4,916,896	114	54,356	9,346
N40	3,890,515	173	43,612	14,541
N50	2,846,712	252	34,871	21,015
N60	2,231,948	354	27,450	29,153
N70	1,533,080	495	20,793	39,687
N80	976,101	702	14,339	54,194
N90	417,397	1,098	7,235	78,238
Total length	2,584,751,267	–	2,519,752,723	–
Total number ≥100 bp	–	194,889	–	338,730
Total number ≥2,000 bp	–	14,815	–	115,749

Note

N50 size is a weighted median statistic indicating that 50% of the entire assembly resides in contigs/scaffolds of a length at least X. N10–N90 are similarly defined.

### 2.6 SNP densities

To check the distribution pattern of SNPs in the genomes, we adopted a method that was described by Hacquard *et al*. (Hacquard et al., 2013). To estimate the distributions of the high‐ and low‐SNP densities, we fitted a two‐component mixture model to the observed SNP densities using the expectation‐maximization (EM) algorithm (function normalmixEM, R‐package mixtools). SNP densities were obtained via a sliding window of 200 kb, at steps of 2 kb, in scaffolds with lengths longer than 300 kb. To identify regions with high‐ and low‐SNP densities, a two‐state hidden Markov model (HMM) was fitted on the 200‐kb SNP densities using the EM algorithm, and the posterior state sequence was computed via the Viterbi algorithm (function fit, package depmixS4).

### 2.7 Genome evolution (gene expansion and contraction families analysis)

The genome and annotation data for *B. taurus, E. caballus, H. sapiens, M. musculus, M. dometica, A. melanoleuca, S. scrofa*, and *C. familiaris* were downloaded from Ensembl (release 73), and the genome and annotation data for *P. hodgsonii, C. hircus, L. vexillifer, B. grunien*, and *C. ferus* were downloaded from the NCBI database. The longest predicted translation product was chosen to represent each gene, and gene models with an open reading frame <150 bp in the genomes were removed. Next, these protein sets were pooled, and self‐to‐self BLASTP (Altschul et al., 1990) was conducted for all of the aforementioned protein sequences with an E‐value of 1e^–5^. Hits with identity values less than 30% and coverage of less than 30% were removed. Then, based on the filtered BLASTP results, orthologous groups were constructed by ORTHOMCL v2.0.9 (Fischer et al., 2011). Phylogenetic tree inference and divergence time estimation was conducted based on fourfold‐degenerate sites of 3,549 single‐copy gene families. Significantly expanded and contracted gene families were identified by CAFÉ (De Bie, Tijl, Demuth, & Hahn, 2006). Molecular evolution analyses were performed using the framework provided by the PAML4.7 package.

Identification of lineage‐specific was rapidly evolving GO categories: First, 9,269 single‐copy gene families of Milu, cow, and human (outgroup) were extracted from orthologous gene clusters identified by OrthoMCL. PRANK (Löytynoja & Goldman, 2008) (v.130410) was used to generate multiple coding sequence alignments with tree file (tree: “((cow, Milu), human);”), and the alignments were filtered using Gblocks (Castresana, 2000) (0.91b). Only genes with ≥300 positions were kept. Furthermore, the resultant 8,768 gene families with reliable codon alignments were annotated to GO terms according to the GO annotations of the human gene (Ensemble release 73) in that gene family. We then calculated the *A* (nonsynonymous substitutions), and *Ka* (nonsynonymous substitutions per nonsynonymous site) values for each remaining gene family using the free‐ratios model and an F3x4 codon frequency model implemented using CODEML in PAML4.7 (Yang, 2007). To determine whether a subset of the categories evolved rapidly in Milu or cow, the *A* values for each GO category were calculated first, and any GO category was containing <15 orthologues was filtered out. For a given GO category, we used the one‐sided Wilcoxon signed‐rank test with a correction for ties as implemented in the R package to calculate the probability of observing an equal or greater number of *A* in Milu or cow. Finally, we extracted the GO categories that met confidence requirements (*p* < 0.025) as Milu or cow lineage‐specific fast evolving GO categories, which were summarized and visualized by REViGO (Supek, Bošnjak, Škunca, & Šmuc, 2011). We also repeated the statistical analysis using *Ka*. The same analysis pipeline was used for TA‐Milu‐human and baiji‐Milu‐human comparisons to obtain lineage‐specific rapidly evolving GO categories in species closely related to Milu and to comprehensively understand the Milu lineage‐specific rapidly evolving GO categories in a different context. Lineage‐specific accelerated evolving GO categories from “biological process” were visualized, and the significantly enriched GO categories in cow, TA, and baiji were consistent with their biological characteristics. This confirmed the robustness of the method (Supporting information Figures S7–S8).

### 2.8 Identification of positive selective genes (PSGS)

The ω (Ka/Ks) ratios of filtered reliable codons in 3,549 single‐copy gene families were calculated using the branch‐site model of CODEML in PAML4.7 (Yang, 2007). Milu was applied as the foreground branch and the others as background branches. We conducted the likelihood ratio test using the χ^2^ statistic to calculate the *p* value and corrected the *p* values for multiple testing by the false discovery rate test with Bonferroni correction to identify PSGs that met the requirements of corrected *p* values <0.01. Statistically significant overrepresented GO terms among these PSGs were identified using BiNGO (Maere, Heymans, & Kuiper, 2005) with the hypergeometric test.

### 2.9 Salinity analyses

A total of 5, 10, 15, 20, 25, 40, 60, 80, 100, 120, 140, 160, 180, and 200 mg of NaCl were weighed, respectively, in separate beakers. A total of 50 ml of distilled water was subsequently mixed with each quantity of NaCl to prepare saline standards. The electric conductivity (EC) value of standard saline was determined using a conductivity meter, and the resultant values were used to generate the *X*‐axis. The saline standard concentration values were used as the *Y*‐axis. A total of 0.5 g of plant materials was weighed in a beaker and mixed with 100 ml of distilled water. After the mixture was heated using an electric stove for 30 min, the resultant solution was strained into a new volumetric flask with 25 ml of distilled water. The solution was stored in a 50‐ml centrifuge tube and was subsequently used to determine EC values.

## 3 RESULTS AND DISCUSSION

### 3.1 Milu genome assembly and annotation

We sequenced and analyzed the Milu genome and performed whole‐genome resequencing for five Milu individuals. The assembled genome (2.58 GB; ~114‐fold coverage) had a scaffold N50 value of 2.85 Mb (Tables 2 and 1). Assembly quality assessment was performed by aligning the transcripts from *Odocoileus virginianus* (white‐tailed deer, WTD) (Malenfant, Davis, Moore, & Coltman, 2013) and *Cervus nippon* (Chinese Sika deer, CSD) (Yao, Zhao, Wang, et al., 2012) to the scaffolds of the Milu (>93.9% and >97.6% coverage, respectively) (Supporting information Table S1) and BUSCO analysis (93.92% of genes were present in our assembly; Supporting information Table S19). We observed that repetitive sequences occupied 39.84% of the whole assembly (Supporting information Tables S2 and S3), and 22,126 protein‐coding genes were predicted by combining de novo and evidence‐based gene predictions (Supporting information Table S4).

**Table 2:** Details of the seven libraries used by the Milu genome sequencing project and statistics of sequencing data of five Milu resequencing sample

Paired‐end insert size	Raw reads				Qualified reads			
	Total reads (Mb)	Total data (Gb)	Read length (bp)	Sequence coverage (*X*)	Total reads (Mb)	Total data (Gb)	Read length (bp)	Sequence coverage (*X*)
De novo geneome								
180 bp	467.92	47.26	101	18.18	458.03	45.22	100	17.39
500 bp	446.41	45.09	101	17.34	431.14	42.28	100	16.26
800 bp	444.19	44.86	101	17.25	400.48	39.36	100	15.14
3 kb	424.34	42.86	101	16.48	388.04	37.32	100	14.35
5 kb	560.61	56.62	101	21.78	510.89	49.86	100	19.18
8 kb	514.64	51.98	101	19.99	455.33	44.41	100	17.08
10 kb	518.48	52.37	101	20.14	419.79	39.12	100	15.05
Total	3,376.59	341.04	101	131.17	3,063.7	297.57	100	114.45

Sample ID	Raw reads				Qualified reads			
	Total reads (Mb)	Total data (Gb)	Read length (bp)	Sequence coverage (*X*)	Total reads (Mb)	Total data (Gb)	Read length (bp)	Sequence coverage (*X*)
Resequencing								
lib2	293.39	29.63	101	11.40	286.60	28.95	101	11.13
lib3	392.32	39.62	101	15.24	362.58	36.62	101	14.09
lib4	382.28	38.61	101	14.85	375.48	37.92	101	14.59
lib5	370.79	37.45	101	14.40	365.98	36.96	101	14.22
lib6	349.72	35.32	101	13.59	342.92	34.63	101	13.32

Note

A total of 341.04 Gb of short reads were generated from seven libraries (7 lanes) to assemble the E. davidianus genome. Insert sizes include paired‐end read lengths. Qualified reads were generated by filtering the low‐quality reads, base‐calling duplicates, and adapter contamination from the raw reads. Coverage was calculated under the assumption of a genome size of 2.6 Gb. Sequence coverage refers to the total length of generated reads.

### 3.2 Relatively large Froh (runs of homozygosity length/genome effective length) of Milu indicates a prolonged reduced Milu population

Milu has been raised in enclosures for more than 1,200 years, with supplementation occurring through the introduction of wild individuals (Li et al., 2011). This resulted in a prolonged genetic bottleneck with reduced genetic diversity. Results generated using the Pairwise Sequentially Markovian Coalescent (PSMC) model (Li & Durbin, 2011) validated this hypothesis (Figure 1d and Supporting information Figure S2). After the Last Glacial Maximum (LGM, ~20 thousand years ago/KYA) (Yokoyama et al., 2000), it is likely that the Milu suffered from the effects of climate change, overhunting and/or habitat loss. Indeed, Milu populations diminished, and there was a tendency toward continuous decreases. This is further evidenced by fossil records and associated literary records (Cao, 2005). Reduced population sizes increase the opportunity for inbreeding. The protracted existence of small populations along with more recent declines resulted in high levels of Milu inbreeding. When related individuals mate, the offspring carry long stretches of a homozygous genome. Thus, the detection of runs of homozygosity (ROH) is a practical approach for estimating inbreeding at the individual level (Kim et al., 2013; Zhou et al., 2014) (Supporting information Table S5). Using SNP genotypes and SNP variants from WGS surveys, Garbe, Prakapenka, Tan, and Da (2016) found that specific populations of Giant Pandas had very high (~ 0.20) inbreeding coefficients (Garbe et al., 2016). We observed that the Froh (ROH length/genome effective length) of the Milu ranged from 0.11 to 0.16 when compared with 34 giant panda genomes (Zhao et al., 2013), 18 polar bear genomes (Liu et al., 2014), and eight crested ibis (Li et al., 2014) genomes. These values are much higher than those for the panda (from 0.04 to 0.10) and polar bear (from 0.004 to 0.064), both of which are less prone to inbreeding. However, the Milu Froh values are lower than those exhibited by the previously critically endangered crested ibis (0.19 for the Milu and 0.32 for the crested ibis), which experienced a more recent and severe genetic bottleneck (Li et al., 2014) (Figure 1e). Length distribution of ROH also provides information about the timing of major inbreeding events. Long ROH is most likely derived from a recent ancestor; shorter stretches are more likely derived from a more distant ancestor (Curik, Ferenčaković, & Sölkner, 2014). As revealed in Figure 1f, the Milu has a medium average ROH length when compared with the crested ibis, the panda, and the polar bear. The crested ibis contains an elongated ROH (longer than 1 M), which is consistent with the fact that current crested ibis populations are derived from seven individuals approximately 40 years ago (Li et al., 2014). The Milu harbor an increased average ROH length compared with the panda and polar bear; however, this value is shorter than that observed for the crested ibis. Given the historical facts regarding the species and the results of the Froh analysis using unrelated individuals (only one pair 2nd degree relatedness in total 10 pairs, Table 3and Supporting information Table S6), the 77 founders of the wild Milu populations in China (Figure 1c) might be far more genetically diverse than the 7 founders of the crested ibis. However, the de novo assembly of the Milu genome had several mis‐assemblies that provide "breaks" in the longer Froh runs than expected.

**Table 3:** The relatedness of five resequencing individuals in this study

	lib2	lib3	lib4	lib5	lib6
lib2	–	**0.13076**	*0*.*03487*	*0*.*02779*	*−0*.*04447*
lib3	–	–	*0*.*04161*	*0*.*10389*	*0*.*03868*
lib4	–	–	–	*−0*.*05938*	*−0*.*02875*
lib5	–	–	–	–	*0*.*01574*
lib6	–	–	–	–	–

Note

The bold value: 2nd Degree; The italic value: Unrelated.

### 3.3 Increased genetic diversity and a low percentage of deleterious variants reveal a reduction in inbreeding depression

**Figure 2:**
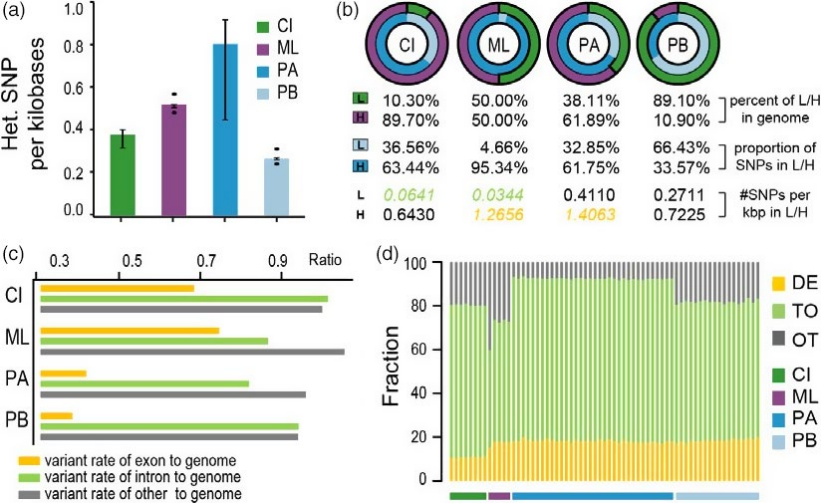
Genetic diversity of the Milu and other animals. (a) Box plot of heterozygosity for Milu, crested ibis, panda, and polar bear individuals. Only heterozygous SNPs were included. CI, Crested Ibis; ML, Milu; PA: Panda; PB: Polar bear. (b) Bias distribution of SNPs in animal genomes. Each circle denotes a single species as in (a). L, low‐SNP density region; H, high‐SNP density region; kbp, kilobase; the proportion of the total length of L and H regions in the whole genome is represented in green and purple; the percentage of SNPs in the L and H regions to the total SNP number in both L and H regions are light blue and blue. (c) Ratio of heterozygosity in each genomic element. The genomes were subdivided into three regions—exons, introns, and other (regions that were neither exons nor introns). Heterozygosity in each type of genomic element was compared to heterozygosity of the whole genome. (d) Classification of missense variants. DE: deleterious; TO: Tolerated; and OT: Other

Another major threat to small and endangered populations involves the loss of genetic diversity (Frankham, 2005; Steiner, Putnam, Hoeck, & Ryder, 2013). Small populations are susceptible to genetic drift and fixation, and these phenomena can be accelerated by inbreeding (Keller & Waller, 2002; Saccheri et al., 1998; Steiner et al., 2013). We observed that genetic diversity was lower in the Milu than in the panda, with a heterozygosity rate of 0.51 per kilobase pair in the Milu, versus 1.32 per kilobase pair in the panda (Supporting information Table S7). Comparison with other endangered animals that experience, or have experienced, ongoing or recent population bottlenecks indicated that this value was similar to that of the mountain gorilla (Xue et al., 2015) (*Gorilla beringei beringei* heterozygosity rate: 0.64 × 10^–3^) but slightly higher than that of the crested ibis (*Nipponia nippon*, heterozygosity rate: 0.36 × 10^–3^, Figure 2a), the Chinese alligator (Wan et al., 2013) (*Alligator sinensis*, heterozygosity rate: 0.15 × 10^–3^), and the baiji (Zhou et al., 2013) (*Lipotes vexillifer*, heterozygosity rate: 0.12 × 10^–3^). Also, patterns of SNP density distributions were explored by fitting a two‐component mixture model to the observed SNP densities using the expectation‐maximization algorithm (Hacquard et al., 2013) (Figure 2b and Supporting information Tables S8–S11). Half of the Milu genome harbors less than 5% of the called SNPs, and the mean heterozygosity of these low‐SNP density regions was 0.03 per kilobase, a value that was similar to that observed in the crested ibis but much lower than those observed in the panda and polar bear. The lower proportion of SNPs also suggests that they were inherited by descent rather than being ancestral runs of homozygosity. But, given that most of five re‐sequenced Milu individuals were unrelated compared to the situation on published crested ibis (Table 3 and Supporting information Table S6), this finding might reflect a more recent inbreeding history concerning the Milu and crested ibis. However, the mean heterozygosity in the other half of the Milu genome was 1.26 per kilobase, which was similar to that observed in the panda but higher than that observed in the crested ibis, indicating a stronger sign of increased diversity in the recovered Milu population than the crested ibis population. Generally, the occurrence of heterozygosity in exons is reduced due to selective constraints (Li et al., 2014). However, the ratio of exon heterozygosity to genome heterozygosity in the Milu and crested ibis is higher than that observed for the panda and polar bear (Figure 2c and Supporting information Table S12). There are two possible explanations for this finding. First, it is possible that the Milu and crested ibis experienced a slower rate of loss of genetic diversity in exons during inbreeding. Second, a rapid increase in the diversity of exons in recovered Milu and crested ibis populations, following the occurrence of severe genetic bottlenecks, may have resulted in greater genetic diversity in these genetic regions.

Inbreeding depression is a major force affecting the evolution and viability of small populations in captive breeding and restoration programs (Keller & Waller, 2002; Saccheri et al., 1998; Steiner et al., 2013). Deleterious mutations tend to accumulate in associated populations due to reduced selective strength (Saccheri et al., 1998; Steiner et al., 2013). We observed that the Milu exhibits a relatively low percentage of deleterious variants (average 17.22%) compared to other healthy or recovered populations (Figure 2d and Supporting information Tables S13–S15). This is consistent with low effective population size (Ne) and the occurrence of inbreeding (Xue et al., 2015). In these populations, alleles occur more frequently in the homozygous state, and because deleterious variants are more likely to be pronounced, they are less likely to persist in the population (even if recessive) (Xue et al., 2015). Bottlenecks may contribute to purging deleterious mutations associated with inbreeding depression (Estoup et al., 2016; Glémin, 2003). Thus, inbreeding depression can be reduced, or purged, by selection against deleterious alleles (Ballou, 1997). Thus, to the aforementioned hypothesis I, populations such as the Milu that has experienced reduced population sizes for prolonged periods may be less susceptible to future inbreeding depressions because they have been purged of deleterious recessive alleles. Consequently, these populations are more likely to recover from future severe genetic bottlenecks.

### 3.4 Analysis of genes involved in sodium channel and high‐salt diet reveal potential high‐salt diet adaptation

**Figure 3:**
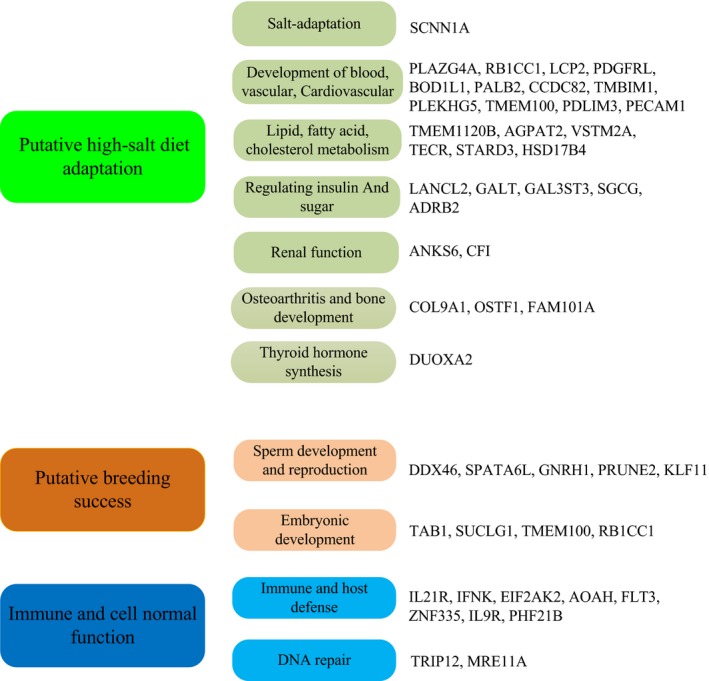
The putative positive selective genes involved in high‐salt diet adaptation and breeding success

**Figure 4:**
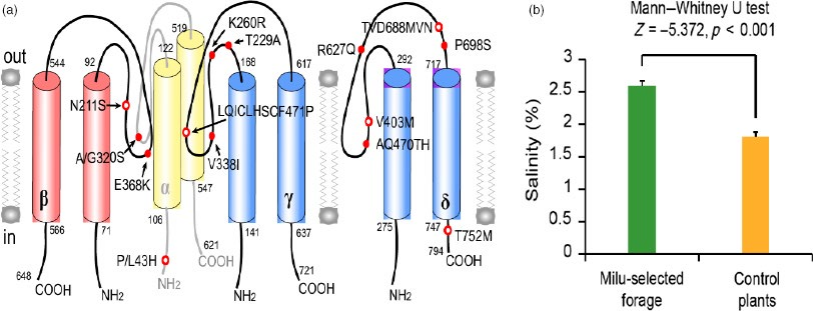
Positive selective pressure signals on genes putatively involved in sodium channels and high‐salt diet. (a) The positive selection analysis on the epithelial sodium channel (ENaC). Red dot, milu‐specific SAPs (single amino acid polymorphisms); red circle, damaging milu‐specific SAPs predicted by PPH2. Red arrows represent the positive selective sites. (b) The salinity of forage plants in Dafeng Milu Natural Reserve

Milu was once widely distributed across coastal (salt water) and inland regions (freshwater). However, long‐term exposure to high‐salt food can lead to higher risks of high blood pressure and cardiovascular disease, and damage to blood vessels, kidneys, and bones in humans (Bath & Butterworth, 1996; Meneton, Jeunemaitre, Wardener, & Macgregor, 2005; Ogihara et al., 2002; Strazzullo, D'Elia, Kandala, & Cappuccio, 2009). Positive natural selection drives an increase in the prevalence of advantageous traits (Sabeti et al., 2006). This form of selection facilitates adaptive molecular evolution and is associated with potential environmental adaptation (Sabeti et al., 2007; Zhang, Nielsen, & Yang, 2005). PSGs of Milu, which are involved in high‐salt diet adaption and breeding success, were identified using the likelihood ratio test implemented in PAML (Yang, 2007) (Figure 3). Interestingly, *SCNN1A* was also under positive selection in Milu. The SCNN1A gene encodes for the α subunit of the epithelial sodium channel (*ENaC*) in vertebrates (Hanukoglu & Hanukoglu, 2016). In most vertebrates, sodium ions are the primary determinant of the osmolarity of the extracellular fluid (Bourque, 2008). *ENaC* allows transfer of sodium ions across the epithelial cell membrane in so‐called tight‐epithelia that have low permeability. The flow of sodium ions across epithelia affects the osmolarity of the extracellular fluid. Thus, *ENaC* plays a central role in the regulation of body fluid and electrolyte homeostasis and consequently affects blood pressure (Rossier, Baker, & Studer, 2015). By scanning Milu‐specific single amino acid polymorphisms (SAPs) in these salt‐sensitive ENaCs, we identified 14 SAPs associated with *SCNN1A*,* SCNN1B*,* SCNN1G*, and *SCNN1D* (Supporting information Figures S3–S6). Two SAPs (N211S and E368K) were under positive selection using the branch‐site model of CODEML in PAML4.7 (Yang, 2007), and six SAPs (including N211S) were predicted to influence channel function, thereby possibly affecting salt‐sensation and sodium absorption (Figure 4a). Upon analysis of gene family numbers, chloride channel activity (*p* = 7.92 × 10^6^) was significantly overrepresented (Supporting information Table S16). These channels are involved in a wide range of biological functions, including blood pressure, epithelial fluid secretion, cell‐volume regulation, and salt sensitivity(Baker, 2000; Jeck, Waldegger, Doroszewicz, Seyberth, & Waldegger, 2004; Jentsch, Maritzen, & Zdebik, 2005; Sheppard & Welsh, 1999). Long‐term high‐salt food can lead to a high risk of high blood pressure, cardiovascular disease, damage to blood vessels, and diabetes in humans (Bath & Butterworth, 1996; Meneton et al., 2005; Ogihara et al., 2001, 2002 ; Strazzullo et al., 2009). In this study, we observed that Milu have signals of positive selection for 29 genes (such as genes encoding for *RB1CC1*,* PLEKHG5*,* PECAM1*,* LANCL2*,* GALT*,* ADRB2*,* STARD3*,* DUOXA2*,* ANKS6*,* CFI,* and *COL9A1*) that are known to be involved in vessel, platelet and cardiovascular development, thyroid hormone synthesis, cholesterol regulation, insulin regulation, and glycemic control (Table 1). For instance, *TMEM100* plays a role during embryonic arterial endothelium differentiation and vascular morphogenesis (Somekawa et al., 2012). Abscisic acid (ABA) is shown to be produced and released by human granulocytes, by insulin‐producing rat insulinoma cells, and by human and murine pancreatic β cells. *LANCL2* is necessary for ABA binding on the cell membrane and activation of the ABA signaling pathway in granulocytes. The natural ligand of *LANCL2*, abscisic acid (ABA), has been identified as a new endogenous mammalian hormone implicated in glycemic control. The trace elements iodine is essential for thyroid gland functioning and thyroid hormone biosynthesis and metabolism (Ingbar & Freinkel, 1955). *DUOXA2* is required for the maturation and the transport from the endoplasmic reticulum to the plasma membrane of functional *DUOX2* and play a role in thyroid hormone synthesis (Grasberger & Refetoff, 2006).

Historically, Milu was widely distributed in the eastern coastal regions of China (Cao, 2005) (Figure 1a), high‐salt and iodine environment. Currently, the largest captive and wild release populations of Milu live in Dafeng Natural Reserve, in the eastern coastal shoal region of China, one of the historically important distribution regions associated with this animal (Figure 1c). The main plant diet of Milu was composed of *Spartina alterniflora*,* Phragmites australis*,* Suaeda glauca*,* Pennisetum alopecuroides*, and *Imperata cylindrica var. major* (Ding, 2017). The salinity of these plants is significantly higher than that for inland plants (Figure 4b and Supporting information Tables S17 and S18). Therefore, to the aforementioned hypothesis II, we concluded that positive signals on genes involved in sodium channels and salt metabolism play an essential role in the adaptation of Milu to a high‐salt diet.

### 3.5 Putative selection pressures on reproduction, development, and immune genes associated with breeding success and fetal development

In this current study, the genomewide analysis revealed that 17 putative genes involved in spermatogenesis, embryogenesis, and innate immune were under positive selection; these genes encode for *DDX46*,* TAB1*,* EIF2AK2*,* AOAH*,* FLT3*,* ZNF335,* and *IL9R* (Figure 3). For instance, *DDX46* encodes a member of the DEAD box protein family, and some members of this family are believed to be involved in embryogenesis, spermatogenesis, and cellular growth and division (Hirabayashi, Hozumi, Higashijima, & Kikuchi, 2013). *TAB1* plays an important role in mammalian embryogenesis (Komatsu et al., 2002). Mutations in *SUCLG1* are the cause of the metabolic disorder fatal infantile lactic acidosis and mitochondrial DNA depletion (Ostergaard et al., 2007). *EIF2AK2* plays a vital role in the innate immune response to viral infection (Irving et al., 2012; Schulz et al., 2010). This finding may imply that potential selection of breeding stocks occurred in the Milu population, thereby supporting the prolonged captive history of the latter. Moreover, the sperm mitochondrial sheath gene family (*p* = 9.85 × 10^−3^) is proportionally expanded in the Milu genome (Supporting information Table S16). The mature sperm tail has several accessory structures, including a mitochondrial sheath, outer dense fibers, and a fibrous sheath (Holstein, 1976). Studies performed on gene knockout mice have proven that precisely regulated mitochondrial sheath formation is critical for sperm motility and fertility (Bouchard et al., 2000; Miki et al., 2004).

Other more pronounced expanded families that were highlighted during this analysis were significantly overrepresented (Supporting information Table S16) by genetic elements on platelet dense granule membranes (*p* = 5.65 × 1,014) and antigen processing and presentation of peptide antigens via MHC class I (*p* = 3.54 × 10^3^). The proteins encoded by these families are essential in eliciting immune system responses against non‐self‐antigens (Neefjes, Jongsma, Paul, & Bakke, 2011). We also identified 26, 25, and 17 lineage‐specific rapidly evolving GO categories that demonstrated a significantly elevated pairwise number of nonsynonymous substitutions (*A*) values in the Milu following Milu‐cow‐human, Milu‐TA‐human, and Milu‐baiji‐human comparisons, respectively. These accelerated evolving GO categories were predominantly found to be involved in responses to immunity, development, DNA repair, and excretion (Supporting information Figures S7 and S8). The DNA repair ability of a cell is vital to the integrity of its genome and thus to the normal functionality of that organism (Alberts et al., 1983). These findings might further illustrate the genetic mechanisms that have enabled the successful environmental adaptation of the Milu.

### 3.6 Milu population recovery and conservation management

The repopulation of the Milu has now deemed a classic example of how a highly endangered species can overcome the threat of extinction. Both external environmental and internal genetics factors have contributed to the successful recovery of this species. External factors include the excellent management of the captive population. Management strategies have ensured that captive populations were protected from natural predators. These strategies have also provided that the captive animals received plenty of food, proper care, and veterinary treatment (where applicable).

However, as revealed by our analyses, the species themselves (internal genetic factors) also inherently contributes to its survival and recovery. Juvenile mortalities are very high in many captive mammals (including ungulate species and inbred cheetah populations), which are most likely caused by inbreeding depression and other factors often associated with inbreeding (O'Brien et al., 1985; Zeng et al., 2007). Thus, the design of the strategies that invoke population recovery of these species is a challenging process. However, the Milu genome exhibits increased genetic diversity and a reduced percentage of deleterious variants indicating a reduction in inbreeding depression (supporting hypothesis I). Genetic mechanisms that have facilitated adaptation to a high‐salt diet and successful reproduction might have also benefited endangered Milu population survival, breeding success, and fetal development (supporting hypothesis II).

The next challenge of Milu population management is the potential for saturation to environmental capacity in maintaining large captive populations, and is when we can reintroduce the animal to historically distribution regions. Large scale of population genetics studies, including several major Milu captive populations (e.g., Hubei Shishou, Beijing Nanhaizi, and Europe populations), will be useful and necessary for these purpose. Using these large‐scale population data, we can evaluate the genetic diversity, population demography, and recent adaptions after genetic bottlenecks (such as selective sweep) of Milu subpopulation, therefore pave the way to the future success of Milu population management.

## CONFLICT OF INTEREST

The authors declare no competing commercial interests.

## AUTHORS’ CONTRIBUTIONS

L.Z. conceived the study, L.Z. headed and H.H. managed the sequencing project, L.Z. and J.D. prepared sequencing data, L.Z., C.D. X.Z., and Z.W. coordinated the bioinformatics activities, L.Z., C.D., X.Z., S.Z., Z.W., G. L, and S.Q. designed experiments and analyzed the data, S.H. and Y.D. participated in project design, L.Z., C.D., Z.Y., and G. L. wrote and edited the manuscript with input from all other authors. All authors have read and approved the manuscript.

### 1 Data Archiving Statement

This whole‐genome shotgun project has been deposited at DDBJ/ENA/GenBank under the accession JRFZ00000000. The version described in this paper is version JRFZ01000000. Correspondence and requests for materials should be addressed to L.Z. (zhulf@ioz.ac.cn).
