# Supplementary material for: Endangered Père David’s deer genome provides insights into population recovering
Source: Evol Appl. 2018 Oct 9;11(10):2040–53. doi: 10.1111/eva.12705 (PMC6231465; doi:10.1111/eva.12705)
Supplement: Supplementary file 1 [file EVA-11-2040-s001.docx]

Supporting Information

Figure S1-S8

Tables S1-S22


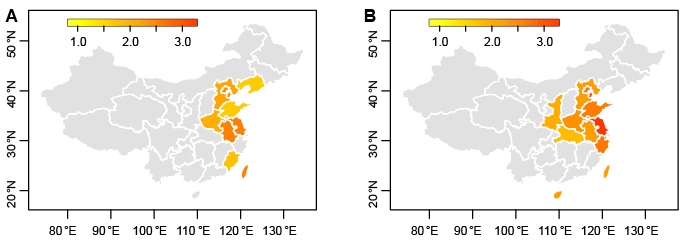


### Fig. S1.

Palaeogeographic distribution of wild Milu in China. The data for Milu fossils were adopted from Cao[^6^](#_ENREF_6), the same as Figure 1; however, the data were split into two datasets according to the geologic time frame: before Holocene (all Pleistocene, **A**) and after Holocene (including Holocene, **B**). The color relates to the density of the fossils in each specific province, and the density was calculated as the number of fossils per million square kilometers.


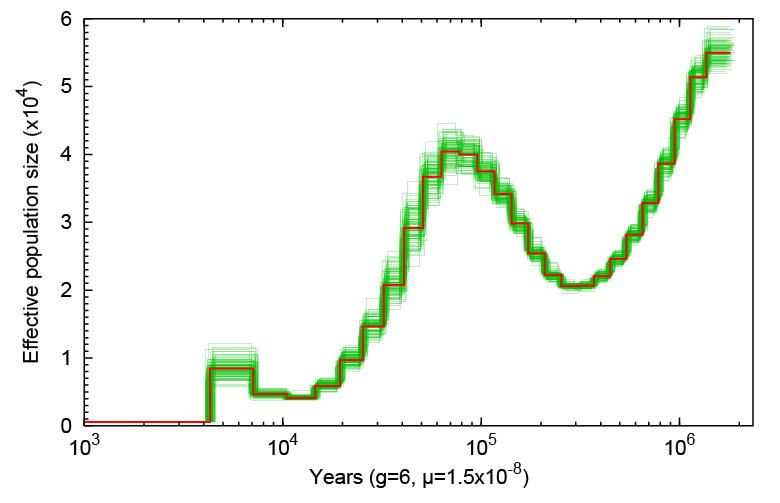


### Fig. S2. Results and bootstrapping for the pairwise sequentially Markovian coalescent for the Milu genome. The red line represents the estimated effective population size, and the 100 thin green curves represent the pairwise sequentially Markovian coalescent estimates for 100 sequences randomly resampled from the original sequence.


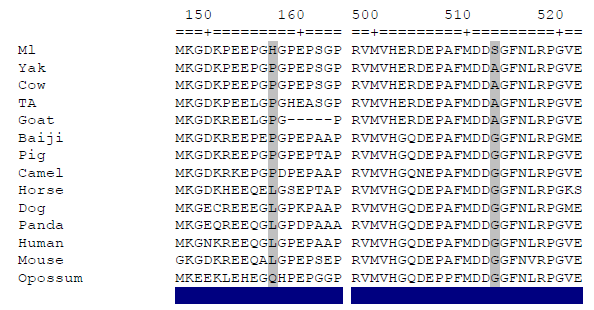


### Fig. S3. Multi-protein alignments from *SCNN1A* gene


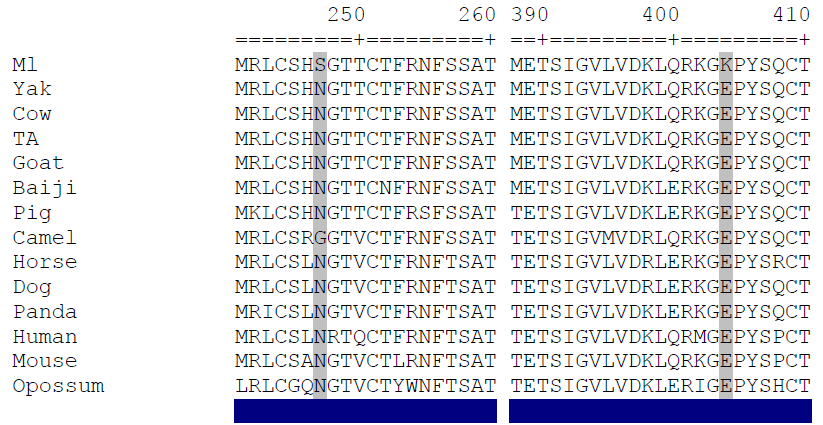


### Fig. S4. Multi-protein alignments from *SCNN1B* gene


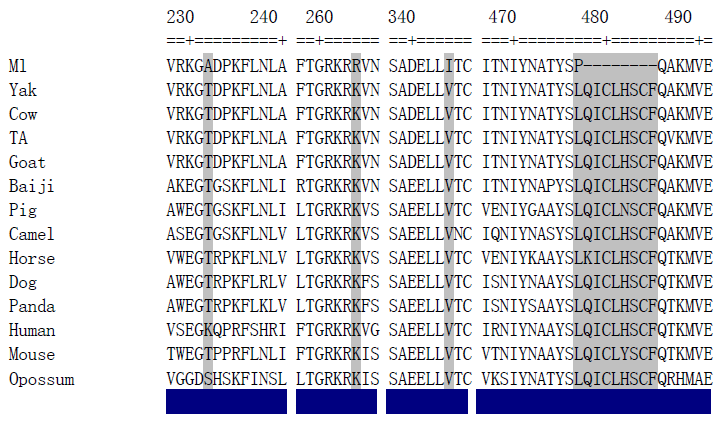


### Fig. S5. Multi-protein alignments from *SCNN1G* gene


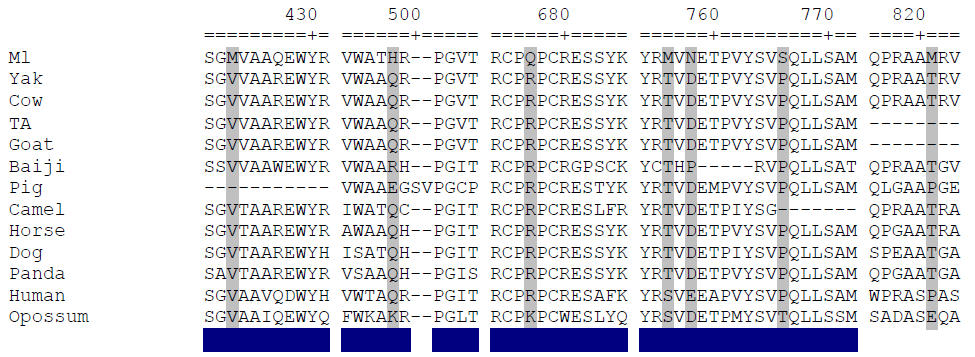


### Fig. S6. Multi-protein alignments from *SCNN1D* gene


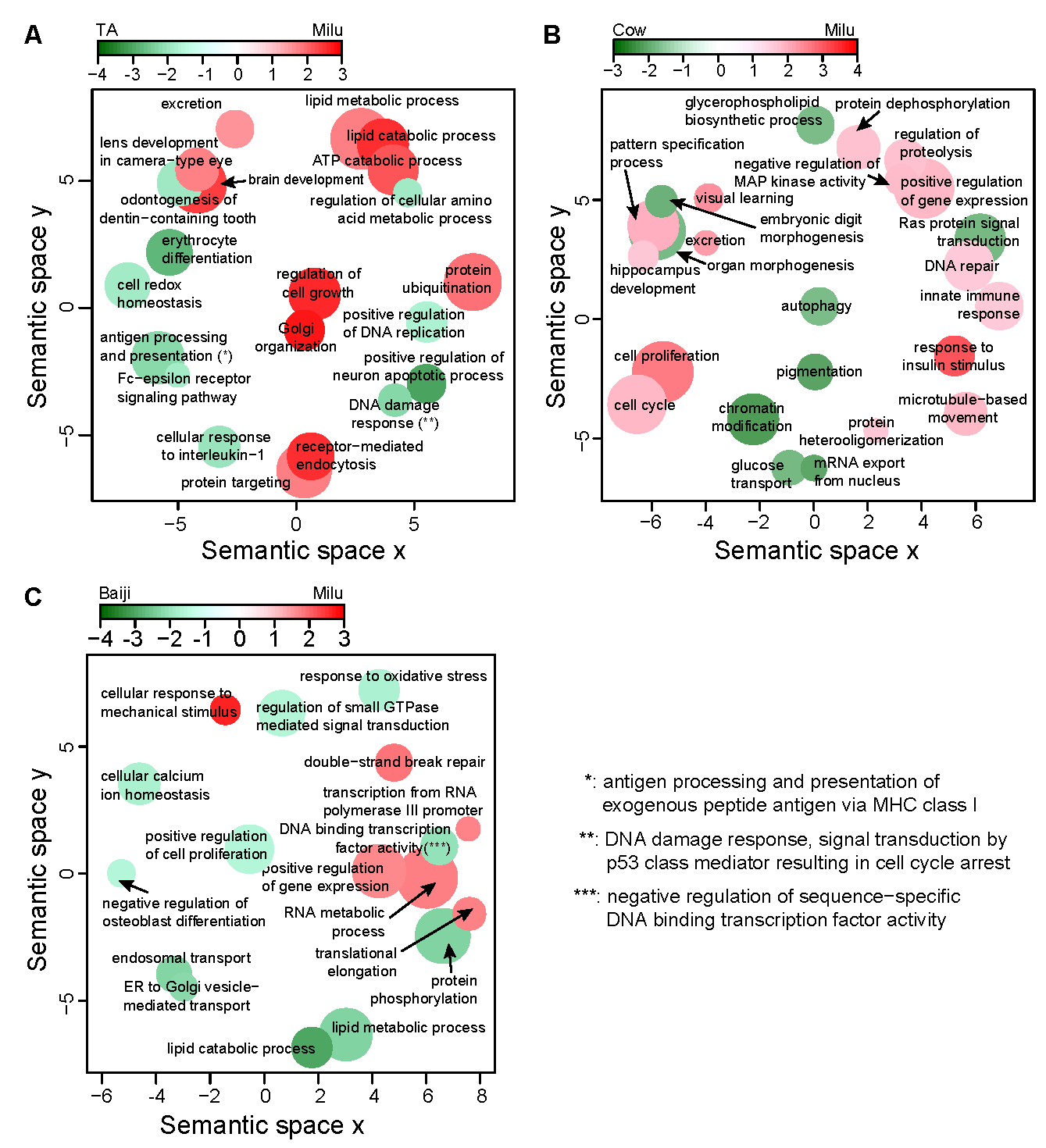


### Fig. S7. Lineage-specific accelerated evolving GO categories of ‘biological process’ using the number of non-synonymous substitutions


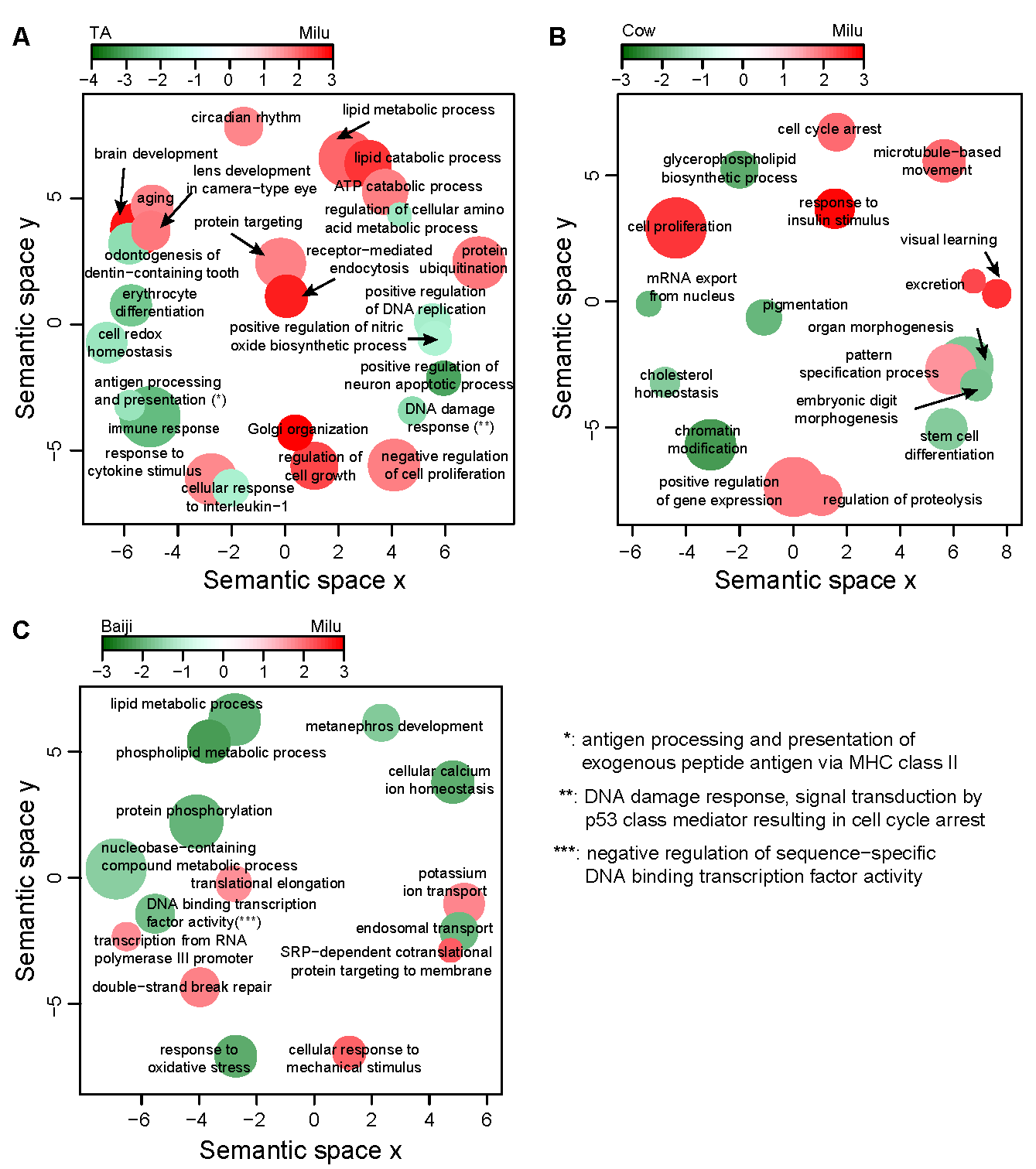


### Fig. S8. Lineage-specific accelerated evolving GO categories of ‘biological process’ using the rate of non-synonymous substitutions

### Table S1. Evaluation of assembly quality of Milu genome by WTD and CSD transcripts

| Dataset | | Number | Total length (bp) | Covered ratio (%) | With >90% sequence in one scaffold | | With >50% sequence in one scaffold | |
| --- | --- | --- | --- | --- | --- | --- | --- | --- |
|  |  |  |  |  | Number | Per cent | Number | Per cent |
| WTD | All | 14,010 | 4,104,760 | 93.92 | 10,798 | 77.07 | 13,658 | 97.49 |
|  | >200bp | 14,010 | 4,104,760 | 93.92 | 10,798 | 77.07 | 13,658 | 97.49 |
|  | >500bp | 677 | 440,104 | 94.53 | 604 | 89.22 | 653 | 96.45 |
|  | >1000b | 28 | 31,462 | 89.44 | 22 | 78.57 | 25 | 89.29 |
| CSD | All | 69,096 | 45,074,746 | 97.62 | 65,257 | 94.44 | 67,696 | 97.97 |
|  | >200bp | 69,096 | 45,074,746 | 97.62 | 65,257 | 94.44 | 67,696 | 97.97 |
|  | >500bp | 22,675 | 31,272,845 | 97.77 | 21,364 | 94.22 | 22,276 | 98.24 |
|  | >1000b | 11,116 | 23,274,981 | 97.83 | 10,431 | 93.84 | 10,935 | 98.37 |

### Table S2. Statistics of the repeat content of the Milu genome

| Type | Repeat size (bp) | % of genome |
| --- | --- | --- |
| TRF | 31,104,798 | 1.20 |
| RepeatMasker | 871,397,796 | 33.71 |
| RepeatProteinMask | 420,237,228 | 16.26 |
| De novo | 944,032,229 | 36.52 |
| Total | 1,097,707,064 | 42.47 |

### Table S3. Statistics of repetitive elements in the Milu genome

| Type | Repbase TEs | | TE proteins | | *De novo* | | Combined TEs | |
| --- | --- | --- | --- | --- | --- | --- | --- | --- |
|  | Length (bp) | %(*) | Length (bp) | %(*) | Length (bp) | %(*) | Length (bp) | %(*) |
| DNA | 34,843,203 | 1.35 | 6,839,054 | 0.26 | 2,131,981 | 0.08 | 35799069 | 1.39 |
| LINE | 603,306,660 | 23.34 | 400,893,596 | 15.51 | 585,805,210 | 22.66 | 751,096,185 | 29.06 |
| SINE | 147,117,448 | 5.69 | 0 | 0.00 | 1,280,353 | 0.05 | 147,983,942 | 5.73 |
| LTR | 93,896,626 | 3.63 | 12,665,343 | 0.49 | 121,876,723 | 4.72 | 191,690,145 | 7.42 |
| Other | 293 | 0.00 | 0 | 0.00 | 0 | 0.00 | 293 | 0.00 |
| Unknown | 156,510 | 0.01 | 0 | 0.00 | 2,352,795 | 0.09 | 2,509,305 | 0.10 |
| Total | 871,397,796 | 33.71 | 420,237,228 | 16.26 | 678,959,441 | 26.27 | 1,029,672,171 | 39.84 |

* percentage in genome. Repbase TE (transposable elements): the result of RepeatMasker based on Repbase; TE proteins: the result of RepeatProteinMask based on Repbase; De novo: repeats found with de novo library; Combined: combined results of Repbase TEs, TE proteins and De novo repeats.

### Table S4. Summary of predicted protein-coding genes and their characteristics

| Gene set | | Number | Average gene length (bp) | Average CDS length (bp) | Average exon per gene (bp) | Average exon length (bp) | Average intron length (bp) |
| --- | --- | --- | --- | --- | --- | --- | --- |
| *De novo* | *AUGUSTUS* | 23,001 | 44,572.77 | 1,411.84 | 8.27 | 170.66 | 5,934.63 |
|  | *GENSCAN* | 50,285 | 32,483.22 | 1,181.69 | 7.1 | 166.34 | 5,128.00 |
|  | *GlimmerHMM* | 30,778 | 11,279.60 | 1,294.16 | 4.53 | 285.54 | 2,826.85 |
| Homolog | *B. taurus* | 25,260 | 19,931.77 | 1,276.80 | 7.18 | 177.90 | 3,020.03 |
|  | *H. sapiens* | 23,231 | 22,569.35 | 1,362.25 | 7.4 | 184.05 | 3,312.90 |
|  | *B. grunniens* | 24,273 | 20,188.42 | 1,246.64 | 7.05 | 176.89 | 3,132.21 |
|  | *C. hircus* | 24,506 | 20,236.98 | 1,230.23 | 6.93 | 177.51 | 3,204.85 |
|  | *P. hodgsonii* | 25,292 | 21,005.32 | 1,328.39 | 7.33 | 181.14 | 3,106.78 |
| EVM | | 23,719 | 25,831.67 | 1,421.76 | 7.9 | 180.06 | 3,539.77 |
| MAKER | | 23,025 | 33,861.89 | 1,521.76 | 8.59 | 177.12 | 4,256.64 |
| Final set | | 22,126 | 38,165.89 | 1,709.79 | 9.66 | 177.08 | 4,210.61 |

### Table S5. ROH length and inbreeding coefficient of each individual

| Species | Individual | ROH(bp) | Froh |
| --- | --- | --- | --- |
| PolarBear | EG01 | 68636467 | 0.0298 |
| PolarBear | EG02 | 146227352 | 0.0635 |
| PolarBear | EG03 | 61034407 | 0.0265 |
| PolarBear | EG04 | 39122678 | 0.0170 |
| PolarBear | EG05 | 78014304 | 0.0339 |
| PolarBear | EG06 | 1019888 | 0.0044 |
| PolarBear | WG01 | 81278639 | 0.0353 |
| PolarBear | WG02 | 68591538 | 0.0298 |
| PolarBear | WG03 | 63009415 | 0.0274 |
| PolarBear | WG04 | 60910721 | 0.0265 |
| PolarBear | WG05 | 71616805 | 0.0311 |
| PolarBear | WG06 | 67861595 | 0.0295 |
| PolarBear | WG07 | 62987561 | 0.0274 |
| PolarBear | WG08 | 52532376 | 0.0228 |
| PolarBear | WG09 | 81723302 | 0.0355 |
| PolarBear | WG10 | 76572633 | 0.0333 |
| PolarBear | WG11 | 63188945 | 0.0274 |
| PolarBear | WG12 | 69964047 | 0.0304 |
| Panda | GP10 | 177807068 | 0.0775 |
| Panda | GP12 | 114958946 | 0.0501 |
| Panda | GP13 | 145531042 | 0.0634 |
| Panda | GP14 | 143031795 | 0.0623 |
| Panda | GP15 | 93885058 | 0.0409 |
| Panda | GP16 | 84316258 | 0.0367 |
| Panda | GP17 | 108121311 | 0.0471 |
| Panda | GP18 | 125273975 | 0.0546 |
| Panda | GP19 | 109951739 | 0.0479 |
| Panda | GP2 | 214985499 | 0.0937 |
| Panda | GP22 | 156407741 | 0.0682 |
| Panda | GP23 | 22316418 | 0.0973 |
| Panda | GP24 | 173084106 | 0.0754 |
| Panda | GP25 | 154281249 | 0.0672 |
| Panda | GP26 | 142210218 | 0.0620 |
| Panda | GP27 | 233062202 | 0.1016 |
| Panda | GP28 | 133908879 | 0.0584 |
| Panda | GP29 | 146767849 | 0.0640 |
| Panda | GP3 | 138425421 | 0.0603 |
| Panda | GP30 | 178562254 | 0.0778 |
| Panda | GP31 | 144622827 | 0.0630 |
| Panda | GP33 | 156866375 | 0.0684 |
| Panda | GP35 | 135522801 | 0.0591 |
| Panda | GP36 | 164890901 | 0.0719 |
| Panda | GP37 | 8236055 | 0.0359 |
| Panda | GP38 | 171568882 | 0.0748 |
| Panda | GP39 | 139472523 | 0.0608 |
| Panda | GP4 | 150299183 | 0.0655 |
| Panda | GP5 | 206504804 | 0.0900 |
| Panda | GP51 | 151086619 | 0.0659 |
| Panda | GP52 | 93204721 | 0.0406 |
| Panda | GP6 | 199999135 | 0.0872 |
| Panda | GP7 | 15586726 | 0.0679 |
| Panda | GP8 | 121266574 | 0.0529 |
| ML | lib2 | 311114813 | 0.1271 |
| ML | lib3 | 269922313 | 0.1103 |
| ML | lib4 | 388144312 | 0.1586 |
| ML | lib5 | 348819516 | 0.1425 |
| ML | lib6 | 379550931 | 0.1551 |
| CrestedIbis | 362M2 | 297423721 | 0.2430 |
| CrestedIbis | 518F2 | 231626527 | 0.1892 |
| CrestedIbis | 550F1 | 299096350 | 0.2444 |
| CrestedIbis | 554M1 | 337093446 | 0.2754 |
| CrestedIbis | 614C2 | 343253729 | 0.2804 |
| CrestedIbis | 617C1 | 385599086 | 0.3150 |
| CrestedIbis | B4-1272 | 301500052 | 0.2463 |
| CrestedIbis | F-C1 | 380701011 | 0.3110 |

### Table S6. The relatedness analyses of the five resequenced Milu individuals (in this study) and eight resequenced crested ibis (published data).


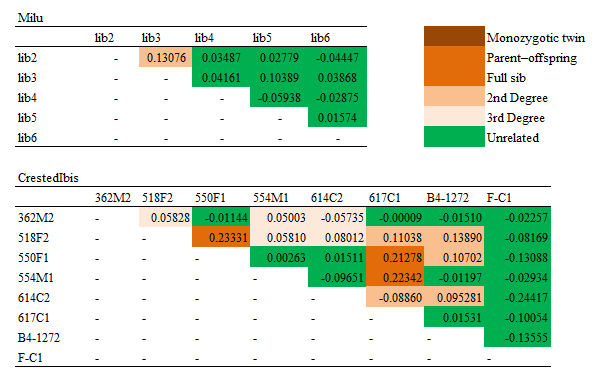


### Table S7. Lambda and mu of each individual under the two-component mixture mode

| Species | Individual | low SNP density | | high SNP density | |
| --- | --- | --- | --- | --- | --- |
|  |  | lambda1 | mu1  (per Kbp) | lambda2 | mu2  (per Kbp) |
| ML | lib2 | 0.515 | 0.032 | 0.485 | 1.163 |
| ML | lib3 | 0.492 | 0.037 | 0.508 | 1.302 |
| ML | lib4 | 0.491 | 0.034 | 0.509 | 1.318 |
| ML | lib5 | 0.503 | 0.035 | 0.497 | 1.286 |
| ML | lib6 | 0.499 | 0.034 | 0.501 | 1.259 |
| CrestedIbis | 362M2 | 0.133 | 0.190 | 0.867 | 0.723 |
| CrestedIbis | 518F2 | 0.106 | 0.046 | 0.894 | 0.666 |
| CrestedIbis | 550F1 | 0.116 | 0.049 | 0.884 | 0.648 |
| CrestedIbis | 554M1 | 0.072 | 0.046 | 0.928 | 0.641 |
| CrestedIbis | 614C2 | 0.147 | 0.044 | 0.853 | 0.585 |
| CrestedIbis | 617C1 | 0.072 | 0.045 | 0.928 | 0.611 |
| CrestedIbis | B4-1272 | 0.093 | 0.044 | 0.907 | 0.628 |
| CrestedIbis | F-C1 | 0.085 | 0.049 | 0.915 | 0.642 |
| Panda | GP10 | 0.371 | 0.342 | 0.629 | 1.262 |
| Panda | GP12 | 0.441 | 0.355 | 0.559 | 1.270 |
| Panda | GP13 | 0.485 | 0.510 | 0.515 | 1.290 |
| Panda | GP14 | 0.407 | 0.363 | 0.593 | 1.277 |
| Panda | GP15 | 0.389 | 0.299 | 0.611 | 1.049 |
| Panda | GP16 | 0.357 | 0.277 | 0.643 | 1.087 |
| Panda | GP17 | 0.387 | 0.384 | 0.613 | 1.327 |
| Panda | GP18 | 0.427 | 0.405 | 0.573 | 1.464 |
| Panda | GP19 | 0.358 | 0.397 | 0.642 | 1.493 |
| Panda | GP2 | 0.425 | 0.599 | 0.575 | 1.782 |
| Panda | GP22 | 0.376 | 0.421 | 0.624 | 1.524 |
| Panda | GP23 | 0.404 | 0.429 | 0.596 | 1.559 |
| Panda | GP24 | 0.370 | 0.398 | 0.630 | 1.505 |
| Panda | GP25 | 0.381 | 0.447 | 0.619 | 1.529 |
| Panda | GP26 | 0.371 | 0.294 | 0.629 | 1.038 |
| Panda | GP27 | 0.417 | 0.483 | 0.583 | 1.549 |
| Panda | GP28 | 0.353 | 0.486 | 0.647 | 1.614 |
| Panda | GP29 | 0.385 | 0.387 | 0.615 | 1.348 |
| Panda | GP3 | 0.286 | 0.438 | 0.714 | 1.621 |
| Panda | GP30 | 0.373 | 0.373 | 0.627 | 1.315 |
| Panda | GP31 | 0.396 | 0.414 | 0.604 | 1.415 |
| Panda | GP33 | 0.440 | 0.285 | 0.560 | 0.993 |
| Panda | GP35 | 0.353 | 0.219 | 0.647 | 0.803 |
| Panda | GP36 | 0.430 | 0.323 | 0.570 | 1.076 |
| Panda | GP37 | 0.322 | 0.474 | 0.678 | 1.578 |
| Panda | GP38 | 0.347 | 0.444 | 0.653 | 1.526 |
| Panda | GP39 | 0.447 | 0.428 | 0.553 | 1.398 |
| Panda | GP4 | 0.299 | 0.405 | 0.701 | 1.518 |
| Panda | GP5 | 0.367 | 0.556 | 0.633 | 1.747 |
| Panda | GP51 | 0.345 | 0.396 | 0.655 | 1.509 |
| Panda | GP52 | 0.337 | 0.553 | 0.663 | 1.769 |
| Panda | GP6 | 0.348 | 0.491 | 0.652 | 1.565 |
| Panda | GP7 | 0.341 | 0.499 | 0.659 | 1.704 |
| Panda | GP8 | 0.423 | 0.400 | 0.577 | 1.310 |
| PolarBear | EG01 | 0.898 | 0.267 | 0.102 | 0.734 |
| PolarBear | EG02 | 0.890 | 0.254 | 0.110 | 0.694 |
| PolarBear | EG03 | 0.890 | 0.264 | 0.110 | 0.717 |
| PolarBear | EG04 | 0.894 | 0.278 | 0.106 | 0.728 |
| PolarBear | EG05 | 0.886 | 0.263 | 0.114 | 0.711 |
| PolarBear | EG06 | 0.897 | 0.299 | 0.103 | 0.770 |
| PolarBear | WG01 | 0.891 | 0.269 | 0.109 | 0.724 |
| PolarBear | WG02 | 0.892 | 0.270 | 0.108 | 0.720 |
| PolarBear | WG03 | 0.884 | 0.267 | 0.116 | 0.693 |
| PolarBear | WG04 | 0.894 | 0.274 | 0.106 | 0.736 |
| PolarBear | WG05 | 0.885 | 0.272 | 0.115 | 0.707 |
| PolarBear | WG06 | 0.891 | 0.272 | 0.109 | 0.720 |
| PolarBear | WG07 | 0.894 | 0.272 | 0.106 | 0.743 |
| PolarBear | WG08 | 0.899 | 0.281 | 0.101 | 0.760 |
| PolarBear | WG09 | 0.885 | 0.267 | 0.115 | 0.703 |
| PolarBear | WG10 | 0.892 | 0.271 | 0.108 | 0.719 |
| PolarBear | WG11 | 0.890 | 0.272 | 0.110 | 0.723 |
| PolarBear | WG12 | 0.886 | 0.268 | 0.114 | 0.703 |

### Table S8. Average lambda and mu of each species under the two-component mixture model

| Species | low SNP density | | high SNP density | |
| --- | --- | --- | --- | --- |
|  | lambda1 | mu1  (per Kbp) | lambda2 | mu2  (per Kbp) |
| CrestedIbis | 0.1030 | 0.0641 | 0.8970 | 0.6430 |
| ML | 0.5000 | 0.0344 | 0.5000 | 1.2656 |
| Panda | 0.3811 | 0.4110 | 0.6189 | 1.4063 |
| PolarBear | 0.8910 | 0.2711 | 0.1090 | 0.7225 |

### Table S9. SNPs in high- and low- SNP density regions identified two-state hidden Markov model

| Species | Individual | #SNPs in high-/low-  density region | SNPs in high | | SNPs in low | |
| --- | --- | --- | --- | --- | --- | --- |
|  |  |  | # | percent | # | percent |
| ML | lib2 | 1,422,426 | 1,354,417 | 95.22% | 68,009 | 4.78% |
| ML | lib3 | 1,662,577 | 1,585,531 | 95.37% | 77,046 | 4.63% |
| ML | lib4 | 1,682,261 | 1,608,472 | 95.61% | 73,789 | 4.39% |
| ML | lib5 | 1,604,578 | 1,527,823 | 95.22% | 76,755 | 4.78% |
| ML | lib6 | 1,590,192 | 1,515,283 | 95.29% | 74,909 | 4.71% |
| CrestedIbis | 362M2 | 743,503 | 332,681 | 44.75% | 410,822 | 55.25% |
| CrestedIbis | 518F2 | 687,842 | 483,307 | 70.26% | 204,535 | 29.74% |
| CrestedIbis | 550F1 | 663,322 | 456,489 | 68.82% | 206,833 | 31.18% |
| CrestedIbis | 554M1 | 684,116 | 437,738 | 63.99% | 246,378 | 36.01% |
| CrestedIbis | 614C2 | 577,257 | 391,340 | 67.79% | 185,917 | 32.21% |
| CrestedIbis | 617C1 | 651,469 | 406,787 | 62.44% | 244,682 | 37.56% |
| CrestedIbis | B4-1272 | 654,981 | 424,639 | 64.83% | 230,342 | 35.17% |
| CrestedIbis | F-C1 | 677,299 | 437,689 | 64.62% | 239,610 | 35.38% |
| Panda | GP10 | 1,962,129 | 1,407,403 | 71.73% | 554,726 | 28.27% |
| Panda | GP12 | 1,846,203 | 1,334,573 | 72.29% | 511,630 | 27.71% |
| Panda | GP13 | 1,925,985 | 1,210,490 | 62.85% | 715,495 | 37.15% |
| Panda | GP14 | 1,926,249 | 1,318,538 | 68.45% | 607,711 | 31.55% |
| Panda | GP15 | 1,617,091 | 1,132,301 | 70.02% | 484,790 | 29.98% |
| Panda | GP16 | 1,699,061 | 1,221,755 | 71.91% | 477,306 | 28.09% |
| Panda | GP17 | 2,046,638 | 1,357,381 | 66.32% | 689,257 | 33.68% |
| Panda | GP18 | 2,155,924 | 1,516,235 | 70.33% | 639,689 | 29.67% |
| Panda | GP19 | 2,334,531 | 1,590,242 | 68.12% | 744,289 | 31.88% |
| Panda | GP2 | 2,712,135 | 1,732,863 | 63.89% | 979,272 | 36.11% |
| Panda | GP22 | 2,359,649 | 1,589,599 | 67.37% | 770,050 | 32.63% |
| Panda | GP23 | 2,340,141 | 1,637,037 | 69.95% | 703,104 | 30.05% |
| Panda | GP24 | 2,328,580 | 1,626,995 | 69.87% | 701,585 | 30.13% |
| Panda | GP25 | 2,369,675 | 1,578,565 | 66.62% | 791,110 | 33.38% |
| Panda | GP26 | 1,621,893 | 1,099,968 | 67.82% | 521,925 | 32.18% |
| Panda | GP27 | 2,346,677 | 1,533,879 | 65.36% | 812,798 | 34.64% |
| Panda | GP28 | 2,580,169 | 1,662,675 | 64.44% | 917,494 | 35.56% |
| Panda | GP29 | 2,083,863 | 1,479,542 | 71.00% | 604,321 | 29.00% |
| Panda | GP3 | 2,718,050 | 1,869,508 | 68.78% | 848,542 | 31.22% |
| Panda | GP30 | 2,051,767 | 1,435,688 | 69.97% | 616,079 | 30.03% |
| Panda | GP31 | 2,165,626 | 1,464,813 | 67.64% | 700,813 | 32.36% |
| Panda | GP33 | 1,452,732 | 995,603 | 68.53% | 457,129 | 31.47% |
| Panda | GP35 | 1,275,286 | 914,738 | 71.73% | 360,548 | 28.27% |
| Panda | GP36 | 1,605,935 | 1,075,846 | 66.99% | 530,089 | 33.01% |
| Panda | GP37 | 2,594,154 | 897,619 | 34.60% | 1,696,535 | 65.40% |
| Panda | GP38 | 2,442,687 | 1,646,990 | 67.43% | 795,697 | 32.57% |
| Panda | GP39 | 2,051,712 | 1,389,706 | 67.73% | 662,006 | 32.27% |
| Panda | GP4 | 2,513,429 | 1,695,005 | 67.44% | 818,424 | 32.56% |
| Panda | GP5 | 2,777,142 | 1,799,749 | 64.81% | 977,393 | 35.19% |
| Panda | GP51 | 2,390,477 | 1,638,761 | 68.55% | 751,716 | 31.45% |
| Panda | GP52 | 2,883,366 | 1,875,189 | 65.03% | 1,008,177 | 34.97% |
| Panda | GP6 | 2,523,851 | 1,725,692 | 68.38% | 798,159 | 31.62% |
| Panda | GP7 | 2,743,383 | 1,906,392 | 69.49% | 836,991 | 30.51% |
| Panda | GP8 | 1,966,098 | 1,329,682 | 67.63% | 636,416 | 32.37% |
| PolarBear | EG01 | 713,565 | 227,006 | 31.81% | 486,559 | 68.19% |
| PolarBear | EG02 | 687,212 | 248,106 | 36.10% | 439,106 | 63.90% |
| PolarBear | EG03 | 711,910 | 246,118 | 34.57% | 465,792 | 65.43% |
| PolarBear | EG04 | 740,086 | 247,830 | 33.49% | 492,256 | 66.51% |
| PolarBear | EG05 | 711,766 | 231,934 | 32.59% | 479,832 | 67.41% |
| PolarBear | EG06 | 787,546 | 261,411 | 33.19% | 526,135 | 66.81% |
| PolarBear | WG01 | 723,686 | 238,028 | 32.89% | 485,658 | 67.11% |
| PolarBear | WG02 | 723,356 | 244,159 | 33.75% | 479,197 | 66.25% |
| PolarBear | WG03 | 717,910 | 244,086 | 34.00% | 473,824 | 66.00% |
| PolarBear | WG04 | 732,006 | 244,985 | 33.47% | 487,021 | 66.53% |
| PolarBear | WG05 | 729,966 | 251,792 | 34.49% | 478,174 | 65.51% |
| PolarBear | WG06 | 727,927 | 245,107 | 33.67% | 482,820 | 66.33% |
| PolarBear | WG07 | 730,429 | 237,213 | 32.48% | 493,216 | 67.52% |
| PolarBear | WG08 | 747,175 | 239,130 | 32.00% | 508,045 | 68.00% |
| PolarBear | WG09 | 719,398 | 242,579 | 33.72% | 476,819 | 66.28% |
| PolarBear | WG10 | 724,160 | 243,465 | 33.62% | 480,695 | 66.38% |
| PolarBear | WG11 | 730,657 | 248,633 | 34.03% | 482,024 | 65.97% |
| PolarBear | WG12 | 720,350 | 247,417 | 34.35% | 472,933 | 65.65% |

### Table S10. SNPs (average) in high- and low- SNP density regions identified two-state hidden Markov model

| Species | #SNPs in high-/low-  density region | SNPs in high | | SNPs in low | |
| --- | --- | --- | --- | --- | --- |
|  |  | # | percent | # | percent |
| CrestedIbis | 667,474 | 421,334 | 0.6344 | 246,140 | 0.3656 |
| ML | 1,592,407 | 1,518,305 | 0.9534 | 74,102 | 0.0466 |
| Panda | 2,188,597 | 1,461,501 | 0.6715 | 727,096 | 0.3285 |
| PolarBear | 726,617 | 243,833 | 0.3357 | 482,784 | 0.6643 |

### Table S11. The features of heterozygous SNP in animals. NMR: Naked mole rats. * The SNP number in CDSs as a percentage of SNPs number in the entire genome. # The SNP rate in CDSs as a percentage of SNPs rate in the entire genome.

| Species | SNP in CDS | Total SNP | Proportion (%)* | %SNP in CDS | %SNP in genome | Ratio (%)# |
| --- | --- | --- | --- | --- | --- | --- |
| Yak | 15,361 | 2,173,164 | 0.71 | 0.051 | 0.089 | 57.30 |
| **Milu** | **14,024** | **1,257,695** | **1.12** | **0.038** | **0.051** | **74.51** |
| panda | 19,115 | 2,682,349 | 0.71 | 0.065 | 0.132 | 49.24 |
| pig | 19,713 | 2,797,447 | 0.70 | 0.068 | 0.118 | 57.63 |
| human | 17,776 | 3,074,097 | 0.58 | 0.025 | 0.099 | 25.25 |
| **NMR** | **15,699** | **1,694,807** | **0.93** | **0.049** | **0.07** | **70.00** |
| **camel** | **19,350** | **1,986,420** | **0.97** | **0.07** | **0.099** | **70.71** |

### Table S12. The features of heterozygous SNP in animals

| Species | SNP in CDS | Total SNP | Proportion (%)* | %SNP in CDS | %SNP in genome | Ratio (%)# |
| --- | --- | --- | --- | --- | --- | --- |
| Yak | 15,361 | 2,173,164 | 0.71 | 0.051 | 0.089 | 57.30 |
| **Milu** | **14,024** | **1,257,695** | **1.12** | **0.038** | **0.051** | **74.51** |
| panda | 19,115 | 2,682,349 | 0.71 | 0.065 | 0.132 | 49.24 |
| pig | 19,713 | 2,797,447 | 0.70 | 0.068 | 0.118 | 57.63 |
| human | 17,776 | 3,074,097 | 0.58 | 0.025 | 0.099 | 25.25 |
| **NMR** | **15,699** | **1,694,807** | **0.93** | **0.049** | **0.07** | **70.00** |
| **camel** | **19,350** | **1,986,420** | **0.97** | **0.07** | **0.099** | **70.71** |

NMR: Naked mole rats. * The SNP number in CDSs as a percentage of SNPs number in the entire genome. # The SNP rate in CDSs as a percentage of SNPs rate in the entire genome.

### Table S13. Functional affections of nsSNPs

| Individual | | lib2 | lib3 | lib4 | lib5 | lib6 |
| --- | --- | --- | --- | --- | --- | --- |
| #nsSNPs | | 10,075 | 11,837 | 11,595 | 11,209 | 11,026 |
| #genes with nsSNPs | | 3,918 | 4,352 | 4,268 | 4,063 | 4,117 |
| SIFT | DELETERIOUS | 1,530 | 2,080 | 2,037 | 2,014 | 1,965 |
|  | DELETERIOUS (%) | 15.19% | 17.57% | 17.57% | 17.97% | 17.82% |
|  | TOLERATED | 4,482 | 6,532 | 6,506 | 6,099 | 6,154 |
|  | NOTSCORED | 1,452 | 1,896 | 1,809 | 1,814 | 1,698 |
|  | - | 2,611 | 1,329 | 1,243 | 1,282 | 1,209 |
| PPH2  (probabilistic  binary  classifier) | deleterious | 1,607 | 1,693 | 1,631 | 1,705 | 1,642 |
|  | deleterious (%) | 15.95% | 14.30% | 14.07% | 15.21% | 14.89% |
|  | neutral | 4,749 | 5,077 | 5,063 | 5,051 | 5,039 |
|  | none | 1,501 | 1,682 | 1,460 | 1,634 | 1,442 |
|  | - | 2,218 | 3,385 | 3,441 | 2,819 | 2,903 |
| PPH2  (qualitative  ternary  classification) | probably damaging | 732 | 787 | 716 | 748 | 750 |
|  | possibly damaging | 935 | 972 | 979 | 1,028 | 956 |
|  | benign | 4,689 | 5,011 | 4,999 | 4,980 | 4,975 |
|  | unknown | 1,501 | 1,682 | 1,460 | 1,634 | 1,442 |
|  | - | 2,218 | 3,385 | 3,441 | 2,819 | 2,903 |

### Table S14. Functional affections of nsSNPs (site is ‘deleterious’ only when both sift and pph2 identifies it as deleterious). *, For PPH2, deleterious nsSNPs include ‘probably damaging’ and ‘possibly damaging’

| Individual | | lib2 | lib3 | lib4 | lib5 | lib6 |
| --- | --- | --- | --- | --- | --- | --- |
| #nsSNPs | | 10,075 | 11,837 | 11,595 | 11,209 | 11,026 |
| #genes with nsSNPs | | 3,918 | 4,352 | 4,268 | 4,063 | 4,117 |
| deleterious* | PPH2+SIFT | 571 | 753 | 688 | 713 | 706 |
|  | PPH2+SIFT (%) | **5.67%** | **6.36%** | **5.93%** | **6.36%** | **6.40%** |
|  | SIFT | 959 | 1,327 | 1,349 | 1,301 | 1,259 |
|  | PPH2 | 1,096 | 1,006 | 1,007 | 1,063 | 1,000 |
|  | Total | 2,626 | 3,086 | 3,044 | 3,077 | 2,965 |
|  | Total (%) | 26.06% | 26.07% | 26.25% | 27.45% | 26.89% |
| non-deleterious | | 5,126 | 7,449 | 8,751 | 8,551 | 8,132 |

### Table S15. The published genome data used in this study

| Species | Protein ID | | | |
| --- | --- | --- | --- | --- |
|  | SCNN1A | SCNN1B | SCNN1C | SCNN1D |
| Cow | ENSAMEP00000015341 | ENSBTAP00000016301 | ENSBTAP00000013412 | ENSBTAP00000023511 |
| Dog | ENSMODP00000035866 | ENSCAFP00000026101 | ENSCAFP00000026102 | ENSCAFP00000028510 |
| Horse | ENSECAP00000002938 | ENSECAP00000012913 | ENSECAP00000018938 | ENSECAP00000009357 |
| Human | ENSCAFP00000022396 | ENSP00000345751 | ENSP00000300061 | ENSP00000368411 |
| Mouse | ENSMUSP00000080164 | ENSMUSP00000033161 | ENSMUSP00000000221 |  |
| Opossum | ENSSSCP00000000753 | ENSMODP00000008443 | ENSMODP00000008496 | ENSMODP00000031197 |
| Panda | ENSBTAP00000003413 | ENSAMEP00000011984 | ENSAMEP00000011923 | ENSAMEP00000014424 |
| Pig | ENSP00000353292 | ENSSSCP00000023321  ENSSSCP00000008364 | ENSSSCP00000008365 | ENSSSCP00000024965 |
| Baiji* | 602718632 | 602729229 | 602729227  602729225 | 602727152 |
| Camel* | 743724234 | 743730382 | 743730384 | 743724388 |
| Goat* | 548468433 | 548520170, 548520174 | 548520166 | 548499240 |
| TA* | 556724351 | 556760089 | 556760091 | 556760091 |
| Yak* | 555991554 | 555987059 | 555987061 | 555987061 |

* NCBI GI Accession number

### Table S16. GO enrichment analysis of gene models found in the expanded gene families of Milu

| GO ID | GO term | GO class | GO level | #Gene | Adjusted  *P*-value |
| --- | --- | --- | --- | --- | --- |
| GO:0004984 | olfactory receptor activity | MF | 5 | 136 | 3.29E-65 |
| GO:0004930 | G-protein coupled receptor activity | MF | 5 | 151 | 2.46E-57 |
| GO:0004888 | transmembrane signaling receptor activity | MF | 4 | 153 | 2.90E-48 |
| GO:0050911 | detection of chemical stimulus involved in sensory perception of smell | BP | 6 | 111 | 2.00E-47 |
| GO:0004872 | receptor activity | MF | 2 | 161 | 3.61E-46 |
| GO:0007186 | G-protein coupled receptor signaling pathway | BP | 5 | 126 | 2.25E-35 |
| GO:0016021 | integral to membrane | CC | 4 | 202 | 3.83E-23 |
| GO:0031224 | intrinsic to membrane | CC | 3 | 204 | 5.34E-23 |
| GO:0071944 | cell periphery | CC | 3 | 173 | 9.83E-18 |
| GO:0005886 | plasma membrane | CC | 3 | 168 | 4.95E-17 |
| GO:0042626 | ATPase activity, coupled to transmembrane movement of substances | MF | 5 | 29 | 6.26E-17 |
| GO:0044425 | membrane part | CC | 2 | 208 | 2.15E-14 |
| GO:0031088 | platelet dense granule membrane | CC | 7 | 11 | 5.65E-14 |
| GO:0007166 | cell surface receptor signaling pathway | BP | 4 | 128 | 2.49E-13 |
| GO:0016503 | pheromone receptor activity | MF | 6 | 15 | 1.89E-12 |
| GO:0006200 | ATP catabolic process | BP | 10 | 26 | 2.85E-12 |
| GO:0042221 | response to chemical stimulus | BP | 3 | 121 | 8.06E-11 |
| GO:0030667 | secretory granule membrane | CC | 6 | 13 | 2.65E-09 |
| GO:0006414 | translational elongation | BP | 6 | 18 | 1.71E-08 |
| GO:0004523 | ribonuclease H activity | MF | 9 | 9 | 2.99E-08 |
| GO:0030911 | TPR domain binding | MF | 5 | 8 | 7.38E-08 |
| GO:0030235 | nitric-oxide synthase regulator activity | MF | 3 | 8 | 7.38E-08 |
| GO:0003964 | RNA-directed DNA polymerase activity | MF | 7 | 9 | 1.09E-07 |
| GO:0015074 | DNA integration | BP | 6 | 9 | 1.09E-07 |
| GO:0017111 | nucleoside-triphosphatase activity | MF | 7 | 51 | 1.74E-07 |
| GO:0016887 | ATPase activity | MF | 8 | 34 | 2.09E-07 |
| GO:0009207 | purine ribonucleoside triphosphate catabolic process | BP | 9 | 43 | 2.67E-07 |
| GO:0003746 | translation elongation factor activity | MF | 6 | 12 | 5.60E-07 |
| GO:0016020 | membrane | CC | 2 | 228 | 7.03E-07 |
| GO:0006278 | RNA-dependent DNA replication | BP | 7 | 9 | 3.19E-06 |
| GO:0005254 | chloride channel activity | MF | 8 | 13 | 7.92E-06 |
| GO:0097159 | organic cyclic compound binding | MF | 3 | 9 | 0.000101 |
| GO:0045429 | positive regulation of nitric oxide biosynthetic process | BP | 5 | 8 | 0.000131 |
| GO:0035605 | peptidyl-cysteine S-nitrosylase activity | MF | 5 | 6 | 0.00018 |
| GO:0035606 | peptidyl-cysteine S-trans-nitrosylation | BP | 8 | 6 | 0.00018 |
| GO:0004365 | glyceraldehyde-3-phosphate dehydrogenase (NAD+) (phosphorylating) activity | MF | 6 | 6 | 0.000309 |
| GO:0071346 | cellular response to interferon-gamma | BP | 6 | 8 | 0.001354 |
| GO:0045040 | protein import into mitochondrial outer membrane | BP | 6 | 5 | 0.002157 |
| GO:0002474 | antigen processing and presentation of peptide antigen via MHC class I | BP | 5 | 6 | 0.003543 |
| GO:0016779 | nucleotidyltransferase activity | MF | 5 | 13 | 0.006097 |
| GO:0005736 | DNA-directed RNA polymerase I complex | CC | 6 | 4 | 0.007529 |
| GO:0097226 | sperm mitochondrial sheath | CC | 4 | 3 | 0.009848 |
| GO:0004190 | aspartic-type endopeptidase activity | MF | 7 | 9 | 0.00986 |

### Table S17. The salinity of major forage plants of Milu

| Main forage plants | Salinity（%） | Location |
| --- | --- | --- |
| Spartina alterniflora | 3.946204 | Jiangsu Dafeng Milu Nature Reserve |
| *Spartina alterniflora* | 2.990895 | Jiangsu Dafeng Milu Nature Reserve |
| *Spartina alterniflora* | 3.215674 | Jiangsu Dafeng Milu Nature Reserve |
| *Spartina alterniflora* | 2.505578 | Jiangsu Dafeng Milu Nature Reserve |
| *Spartina alterniflora* | 3.26676 | Jiangsu Dafeng Milu Nature Reserve |
| *Spartina alterniflora* | 3.271868 | Jiangsu Dafeng Milu Nature Reserve |
| *Spartina alterniflora* | 2.875952 | Jiangsu Dafeng Milu Nature Reserve |
| *Spartina alterniflora* | 2.620522 | Jiangsu Dafeng Milu Nature Reserve |
| *Spartina alterniflora* | 3.156925 | Jiangsu Dafeng Milu Nature Reserve |
| *Spartina alterniflora* | 3.445561 | Jiangsu Dafeng Milu Nature Reserve |
| *Spartina alterniflora* | 3.923215 | Jiangsu Dafeng Milu Nature Reserve |
| *Spartina alterniflora* | 3.614145 | Jiangsu Dafeng Milu Nature Reserve |
| *Spartina alterniflora* | 2.411069 | Jiangsu Dafeng Milu Nature Reserve |
| *Spartina alterniflora* | 2.86318 | Jiangsu Dafeng Milu Nature Reserve |
| *Spartina alterniflora* | 4.040713 | Jiangsu Dafeng Milu Nature Reserve |
| *Spartina alterniflora* | 2.270583 | Jiangsu Dafeng Milu Nature Reserve |
| *Spartina alterniflora* | 3.746968 | Jiangsu Dafeng Milu Nature Reserve |
| *Spartina alterniflora* | 1.82358 | Jiangsu Dafeng Milu Nature Reserve |
| *Spartina alterniflora* | 2.829975 | Jiangsu Dafeng Milu Nature Reserve |
| *Spartina alterniflora* | 3.052199 | Jiangsu Dafeng Milu Nature Reserve |
| *Spartina alterniflora* | 2.704814 | Jiangsu Dafeng Milu Nature Reserve |
| *Spartina alterniflora* | 2.635848 | Jiangsu Dafeng Milu Nature Reserve |
| *Spartina alterniflora* | 2.255257 | Jiangsu Dafeng Milu Nature Reserve |
| *Spartina alterniflora* | 2.898941 | Jiangsu Dafeng Milu Nature Reserve |
| *Spartina alterniflora* | 2.663945 | Jiangsu Dafeng Milu Nature Reserve |
| *Spartina alterniflora* | 2.855518 | Jiangsu Dafeng Milu Nature Reserve |
| *Spartina alterniflora* | 2.944918 | Jiangsu Dafeng Milu Nature Reserve |
| *Spartina alterniflora* | 3.162034 | Jiangsu Dafeng Milu Nature Reserve |
| *Spartina alterniflora* | 2.909158 | Jiangsu Dafeng Milu Nature Reserve |
| *Spartina alterniflora* | 4.602659 | Jiangsu Dafeng Milu Nature Reserve |
| *Spartina alterniflora* | 3.879792 | Jiangsu Dafeng Milu Nature Reserve |
| *Spartina alterniflora* | 3.792946 | Jiangsu Dafeng Milu Nature Reserve |
| *Spartina alterniflora* | 3.187577 | Jiangsu Dafeng Milu Nature Reserve |
| *Spartina alterniflora* | 2.7559 | Jiangsu Dafeng Milu Nature Reserve |
| *Spartina alterniflora* | 3.210565 | Jiangsu Dafeng Milu Nature Reserve |
| *Spartina alterniflora* | 2.927038 | Jiangsu Dafeng Milu Nature Reserve |
| *Spartina alterniflora* | 3.10073 | Jiangsu Dafeng Milu Nature Reserve |
| *Spartina alterniflora* | 2.232268 | Jiangsu Dafeng Milu Nature Reserve |
| *Spartina alterniflora* | 2.324223 | Jiangsu Dafeng Milu Nature Reserve |
| *Spartina alterniflora* | 3.039427 | Jiangsu Dafeng Milu Nature Reserve |
| *Phragmites australis* | 2.671608 | Jiangsu Dafeng Milu Nature Reserve |
| *Phragmites australis* | 1.560487 | Jiangsu Dafeng Milu Nature Reserve |
| *Phragmites australis* | 2.339549 | Jiangsu Dafeng Milu Nature Reserve |
| *Phragmites australis* | 1.402121 | Jiangsu Dafeng Milu Nature Reserve |
| *Phragmites australis* | 2.651174 | Jiangsu Dafeng Milu Nature Reserve |
| *Phragmites australis* | 2.058576 | Jiangsu Dafeng Milu Nature Reserve |
| *Phragmites australis* | 2.492807 | Jiangsu Dafeng Milu Nature Reserve |
| *Phragmites australis* | 2.388081 | Jiangsu Dafeng Milu Nature Reserve |
| *Phragmites australis* | 3.345943 | Jiangsu Dafeng Milu Nature Reserve |
| *Phragmites australis* | 2.365092 | Jiangsu Dafeng Milu Nature Reserve |
| *Phragmites australis* | 3.465995 | Jiangsu Dafeng Milu Nature Reserve |
| *Phragmites australis* | 2.73802 | Jiangsu Dafeng Milu Nature Reserve |
| *Erigeron annuus* | 1.540053 | Jiangsu Dafeng Milu Nature Reserve |
| *Erigeron annuus* | 1.670322 | Jiangsu Dafeng Milu Nature Reserve |
| *Erigeron annuus* | 2.615413 | Jiangsu Dafeng Milu Nature Reserve |
| *Erigeron annuus* | 1.746951 | Jiangsu Dafeng Milu Nature Reserve |
| *Erigeron annuus* | 2.434058 | Jiangsu Dafeng Milu Nature Reserve |
| *Erigeron annuus* | 1.565596 | Jiangsu Dafeng Milu Nature Reserve |
| *Erigeron annuus* | 2.06113 | Jiangsu Dafeng Milu Nature Reserve |
| *Erigeron annuus* | 1.782712 | Jiangsu Dafeng Milu Nature Reserve |
| *Imperata cylindrica* | 1.8031459 | Jiangsu Dafeng Milu Nature Reserve |
| *Imperata cylindrica* | 1.718854 | Jiangsu Dafeng Milu Nature Reserve |
| *Imperata cylindrica* | 1.7724943 | Jiangsu Dafeng Milu Nature Reserve |
| *Imperata cylindrica* | 1.744397 | Jiangsu Dafeng Milu Nature Reserve |
| *Imperata cylindrica* | 1.9104265 | Jiangsu Dafeng Milu Nature Reserve |
| *Imperata cylindrica* | 1.7060825 | Jiangsu Dafeng Milu Nature Reserve |
| *Imperata cylindrica* | 1.7801572 | Jiangsu Dafeng Milu Nature Reserve |
| *Imperata cylindrica* | 1.8567862 | Jiangsu Dafeng Milu Nature Reserve |
| *Imperata cylindrica* | 2.357429 | Jiangsu Dafeng Milu Nature Reserve |
| *Imperata cylindrica* | 2.0585759 | Jiangsu Dafeng Milu Nature Reserve |
| *Pennisetum alopecuroides* | 2.382972 | Jiangsu Dafeng Milu Nature Reserve |
| *Pennisetum alopecuroides* | 1.9410781 | Jiangsu Dafeng Milu Nature Reserve |
| *Pennisetum alopecuroides* | 2.4876983 | Jiangsu Dafeng Milu Nature Reserve |
| *Pennisetum alopecuroides* | 2.8223116 | Jiangsu Dafeng Milu Nature Reserve |
| *Pennisetum alopecuroides* | 1.9640668 | Jiangsu Dafeng Milu Nature Reserve |
| *Pennisetum alopecuroides* | 2.5719902 | Jiangsu Dafeng Milu Nature Reserve |
| *Pennisetum alopecuroides* | 2.5234585 | Jiangsu Dafeng Milu Nature Reserve |
| *Pennisetum alopecuroides* | 1.6345621 | Jiangsu Dafeng Milu Nature Reserve |
| *Pennisetum alopecuroides* | 2.357429 | Jiangsu Dafeng Milu Nature Reserve |
| *Pennisetum alopecuroides* | 1.795483 | Jiangsu Dafeng Milu Nature Reserve |
| *Pennisetum alopecuroides* | 2.1556393 | Jiangsu Dafeng Milu Nature Reserve |

### Table S18. The salinity of control plants

| Control plants | Salinity（%） | Location |
| --- | --- | --- |
| *Setaria viridis* | 1.613125 | Nanjing, Jiangsu |
| *Setaria viridis* | 2.0063542 | Nanjing, Jiangsu |
| *Setaria viridis* | 2.1990625 | Nanjing, Jiangsu |
| *Setaria viridis* | 1.5297917 | Nanjing, Jiangsu |
| *Setaria viridis* | 1.9933333 | Nanjing, Jiangsu |
| *Setaria viridis* | 1.4178125 | Nanjing, Jiangsu |
| *Setaria viridis* | 1.4204167 | Nanjing, Jiangsu |
| *Setaria viridis* | 1.7225 | Nanjing, Jiangsu |
| *Setaria viridis* | 1.12875 | Nanjing, Jiangsu |
| *Setaria viridis* | 1.87875 | Nanjing, Jiangsu |
| *Setaria viridis* | 1.2172917 | Nanjing, Jiangsu |
| *Setaria viridis* | 1.3605208 | Nanjing, Jiangsu |
| *Melia azedarach L* | 2.0454167 | Nanjing, Jiangsu |
| *Melia azedarach L* | 1.3969792 | Nanjing, Jiangsu |
| *Loropetalum chinense var.rubrum* | 1.363125 | Nanjing, Jiangsu |
| *Loropetalum chinense var.rubrum* | 1.0636458 | Nanjing, Jiangsu |
| *Cinnamomum camphora* | 1.863125 | Nanjing, Jiangsu |
| *Cinnamomum camphora* | 1.2120833 | Nanjing, Jiangsu |
| *Dichondra micrantha* | 2.2719792 | Nanjing, Jiangsu |
| *Dichondra micrantha* | 2.6391667 | Nanjing, Jiangsu |
| *Jasminum nudiflorum* | 2.2251042 | Nanjing, Jiangsu |
| *Jasminum nudiflorum* | 1.9021875 | Nanjing, Jiangsu |
| *Jasminum nudiflorum* | 2.1678125 | Nanjing, Jiangsu |
| *Jasminum nudiflorum* | 1.8527083 | Nanjing, Jiangsu |
| *Jasminum nudiflorum* | 2.1652083 | Nanjing, Jiangsu |
| *Jasminum nudiflorum* | 1.7902083 | Nanjing, Jiangsu |
| *Jasminum nudiflorum* | 2.3839583 | Nanjing, Jiangsu |
| *Jasminum nudiflorum* | 1.8891667 | Nanjing, Jiangsu |
| *Jasminum nudiflorum* | 2.0610417 | Nanjing, Jiangsu |
| *Jasminum nudiflorum* | 2.2251042 | Nanjing, Jiangsu |
| *Jasminum nudiflorum* | 1.8214583 | Nanjing, Jiangsu |
| *Jasminum nudiflorum* | 2.0428125 | Nanjing, Jiangsu |

### Table S19. Assessing genome assembly and annotation completeness with single-copy orthologs

|  | Assembly | |
| --- | --- | --- |
|  | Proteins | Percentage (%) |
| Complete Single-Copy BUSCOs | 5,434 | 86.90 |
| Complete Duplicated BUSCOs | 95 | 1.52 |
| Fragmented BUSCOs | 344 | 5.50 |
| Missing BUSCOs | 380 | 6.08 |
| Total BUSCO groups searched | 6,253 | 100.00 |
